# Obesity and Insulin Resistance Alter Neural Processing of Unpleasant, but Not Pleasant, Visual Stimuli in Young Adults

**DOI:** 10.3390/brainsci16010003

**Published:** 2025-12-19

**Authors:** Brittany A. Larsen, Brandon S. Klinedinst, Tovah Wolf, Kelsey E. McLimans, Qian Wang, Parvin Mohammadiarvejeh, Mohammad Fili, Azizi A. Seixas, Auriel A. Willette

**Affiliations:** 1 Department of Psychiatry and Behavioral Sciences, University of Miami Miller School of Medicine, 1120 NW 14th St., Miami, FL 33136, USA; bal184@miami.edu (B.A.L.); azizi.seixas@med.miami.edu (A.A.S.); 2 Department of Neurology, Robert Wood Johnson Medical School, Rutgers University, 125 Paterson St., New Brunswick, NJ 08901, USA; bsk104@ifh.rutgers.edu; 3 TDWOLF Nutrition, 4505 Kenny Rd., #1019, Columbus, OH 43220, USA; tovah@tdwolfnutrition.com; 4 Nutrition and Dietetics Department, Viterbo University, 900 Viterbo Dr., La Crosse, WI 54601, USA; kemclimans@viterbo.edu; 5 Department of Food Science and Human Nutrition, College of Human Sciences, Iowa State University, 2312 Food Sciences Building, 536 Farm House Ln, Ames, IA 50011, USA; wangqian@iastate.edu; 6 University of Maryland Institute for Health Computing, 6116 Executive Blvd., North Bethesda, MD 20852, USA; p.mohammadiarvejeh@umm.edu; 7 Department of Systems Engineering and Operations Research, College of Engineering and Computing, George Mason University, 4400 University Drive, Fairfax, VA 22030, USA; mfili@gmu.edu; 8 Department of Informatics and Health Data Science, University of Miami Miller School of Medicine, 1120 NW 14th St., Miami, FL 33136, USA

**Keywords:** electroencephalography (EEG), emotion, event-related potentials (ERPs), insulin resistance, neurophysiology, obesity, late positive potential (LPP), early posterior negativity (EPN), young adults, emotional processing

## Abstract

**Background/Objectives**: Obesity and insulin resistance (IR) increase the risk of mood disorders, which often manifest during young adulthood. However, neuroelectrophysiological investigations of whether adiposity and IR modify electrocortical activity and emotional processing outcomes remain underexplored, particularly in young adults. Therefore, this study used electroencephalography (EEG) to investigate whether obesity and/or IR moderate the relationships between brain potentials and affective processing in younger adults. **Methods**: Thirty younger adults completed a passive picture-viewing task utilizing the International Affective Picture System while real-time electroencephalography was simultaneously recorded. Two event-related potentials—early posterior negativity (EPN) and late positive potential (LPP)—were quantified. Affective processing parameters included mean valence ratings and stimulus-to-response-onset reaction times in response to unpleasant, pleasant, and neutral images. Body fat percentage and Homeostatic Model Assessment for Insulin Resistance values were measured. Hierarchical moderated regression analysis was utilized to test the interrelationships between brain potentials, adiposity, IR, and affective processing. **Results**: In the Negative−Neutral condition, lean and insulin-sensitive participants gave less negative valence ratings to unpleasant versus neutral images when late-window LPP amplitudes were larger, whereas this relationship was reversed in participants with obesity and absent in those with IR. Contrariwise, neither obesity nor IR moderated LPP responses to affective processing parameters in the Positive−Neutral or Negative−Positive valence conditions. Additionally, obesity and IR did not moderate the links between EPN responses and affective processing parameters in any of the valence conditions. **Conclusions**: Lean, insulin-sensitive young adults showed attenuated affective processing of unpleasant stimuli through stronger neural responses, whereas neural responses to pleasant stimuli did not vary across levels of body fat or IR. These preliminary findings suggest that both obesity and IR increase the vulnerability to mood disorders in young adulthood.

## 1. Introduction

Global adult obesity rates have more than doubled from 7% in 1990 to 16% in 2022 [1]. Obesity increases the risk of mood disorders [2,3], which are often characterized by alterations in emotional processing [4] and most commonly manifest during young adulthood [5]. Obesity has also been linked to alterations in affective processing, namely enhanced automatic negative affective processing [6,7,8] and, to a lesser extent, attenuated automatic positive affective processing [9]. In contrast to these findings, an exploratory meta-analysis comprising 13 studies reported that emotional processing was not significantly impaired in those with obesity compared to controls. However, this meta-analysis pooled studies across all age groups [10]. Nevertheless, a preliminary study likewise did not find a deficit in affective processing in young subjects with obesity; however, this study was exclusive to children [11]. Hence, evidence of whether obesity influences affective processing in young adults remains unclear. Insulin resistance (IR) is another globally prevalent risk factor for mood disorders [12,13]. A recent meta-analysis reported that IR is prevalent in 26.5% of adults worldwide [14]. Insulin, a pancreatic hormone, functions in whole-body glycolytic processes [15]. IR emerges when target tissues are less responsive to insulin stimulation [16]. IR has been consistently correlated with emotional processing impairments across various populations, including children [17], younger adult females with polycystic ovary syndrome [18], and male mice [19]. However, the relationship between IR and affective processing impairments in otherwise healthy younger adults remains underexplored in the prevailing body of literature. Yet, considering that global rates of mood disorders have increased considerably [20], it is crucial to explore whether modifiable risk factors such as excess adiposity and IR could be linked to deficits in affective processing, particularly in an age group when such disorders most commonly emerge.

Neurophysiologically, the neurocircuitry underlying affective processing involves widespread brain regions. Affective processing commences with an automatic, nonconscious response to an emotional stimulus that is signaled by increased amygdala activity [21]. Information about emotional arousal is subsequently relayed from the amygdala and basal ganglia (BG) to the ventrolateral prefrontal cortex (vlPFC), anterior insula (aINS), supplementary motor area (SMA), angular gyrus (AG), and superior temporal gyrus (STG). The vlPFC and aINS evaluate the affective input to determine whether the affective arousal necessitates emotional regulation; the SMA, AG, and STG respectively simulate the motor, somatosensory, and language processes associated with the affective input to enhance an emotional experience. If emotion regulation is required, the vlPFC and aINS further project this information to the dorsolateral PFC (dlPFC), where affect regulation occurs (i.e., downregulation of unpleasant stimuli and upregulation of pleasant stimuli). Subsequently, the dlPFC projects a feedforward signal indirectly through the anterior middle cingulate gyrus or directly back to the amygdala, BG, SMA, AG, and STG. These events, in turn, contribute to the production of a newly regulated emotional state [22]. However, obesity and IR may impair these processes by inducing structural and functional alterations in brain structures that modulate affective processing. For example, greater adiposity has been linked to fewer dopamine striatal D2 receptors [23], which, in turn, was associated with impaired prefrontal metabolism within the orbitofrontal (OFC) and anterior cingulate cortex (ACC) in those with obesity [24]. These neural modifications may reduce prefrontal modulation of affective input from the amygdala and BG, resulting in amplified responses to unpleasant stimuli and enhanced negative affective processing. Furthermore, antagonistic action against dopamine D2/D3 receptors attenuates striatal activity in response to rewarding stimuli [25], which may partially explain why blunted positive affective processing has also been found in subjects with adiposity [9]. Additionally, a neuroimaging study observed blunted activity in the amygdalae of subjects with obesity, which was linked to a greater susceptibility to feelings of negative affectivity [26]. Similar to obesity, IR is also correlated with brain structural and functional alterations. Peripheral IR impedes central signaling, causing central IR [27] and hindering brain glucose metabolism. Chronic central IR engenders progressive brain atrophy [28]. IR-induced atrophy is most pronounced in brain regions that function in affective processing (i.e., PFC, medial temporal lobe, parietal gyri [29]), which may impede emotional processing over time. Central IR has also been associated with impediments in dopamine turnover and mitochondrial dysfunction, which, in turn, increases the risk of mood disorders [30]. These findings collectively suggest that impairments in affective processing among those with obesity and/or IR may stem from diminished top-down modulation of affectivity as a consequence of brain structural and functional impairments induced by these risk factors.

The prevailing empirical evidence has most frequently utilized functional magnetic resonance imaging (fMRI) to investigate if obesity and/or IR alter neural activity and underlying affective processing [17,18,26,31,32]. fMRI provides detailed characterization of hemodynamic responses and, relative to other neuroimaging techniques, has the highest spatial resolution for identifying the precise neuroanatomical origins of neural activity [33]. However, fMRI has several notable limitations, including poor temporal resolution, an inability to capture electrophysiological aspects of brain function [33], and high cost [34]. By contrast, electroencephalography (EEG) is an inexpensive, non-invasive neuroimaging approach that provides millisecond-level temporal dynamics and electrophysiological signatures of ongoing neural activity [35]. EEG has been used extensively in studies of emotional processing (e.g., [36,37,38,39]). Despite this, neuroelectrophysiological investigations of whether adiposity or IR modify the relationships between brain function and affective processing outcomes are remarkably underexplored to date. The paucity of evidence that has investigated this has focused on older adults [40]. Hence, no studies to date have used EEG to investigate whether obesity or IR alter the relationships between neural activity and negative or positive affective processing in young adults. This gap is disconcerting when considering that young adulthood is when mood disorders most frequently manifest [41]. Therefore, exploring whether obesity and/or IR may alter temporal and electrophysiological dynamics of brain function as well as emotional processing outcomes is crucial in this age group to gain a better understanding of how these modifiable risk factors may shape early neural mechanisms implicated in later mood vulnerability.

Electrical activity that is recorded by EEG during a task is utilized to quantify event-related potentials (ERPs), which are voltage fluctuations captured by an electroencephalogram that are induced by a particular event or stimulus [42]. Each ERP component is functionally differentiated according to: (1) polarity (direction of amplitude deflection); (2) scalp distribution; (3) latency (time and duration of electrocortical activity); and (4) sensitivity to particular task-linked manipulations [43]. Two well-studied ERP components—early posterior negativity (EPN) and late positive potential (LPP)—index affective processing [44]. EPN is a negative, occipitotemporal potential that typically reaches a trough between 200–300 milliseconds (ms) post-stimulus exposure. Larger (i.e., more negative) EPN voltage amplitudes reflect an increase in postsynaptic potentials in the extrastriate visual cortex [45] and functionally index early affective categorization and attentional engagement with relevant stimuli [46]. EPN is particularly sensitive to Positively valenced stimuli [47]. LPP is a positive, centroparietal potential that commences 300–400 ms post-stimulus exposure [48,49]. Higher (i.e., more positive) LPP voltage amplitudes reflect an increase in prolonged postsynaptic potentials in the parietal and prefrontal cortices [50]. LPP amplitudes are consistently higher in response to emotional versus neutral stimuli [51,52] and are generally sustained throughout the continuance of stimulus presentation [48]. As a slow-evolving component, LPP is often segmented into three distinct latency windows that represent neural responses during three sequential affective processing stages: (1) automatic emotional response; (2) sustained attention to and ongoing evaluation of emotional stimuli; and (3) prolonged affective processing and/or affect regulation [44,53,54,55,56]. During the early latency window, LPP amplitudes peak in the centroparietal area [56,57,58] and functionally index emotional salience in terms of motivated attention during passive picture-viewing tasks. Early-window LPP amplitudes reflect the level of arousal elicited by emotional stimuli [53,54,56]. In contrast, late-window LPP amplitudes shift to a frontal distribution [56,57,58] and reflect greater processing of affective stimuli relative to neutral during passive picture-viewing tasks [54,55,56]. Late-window LPP amplitudes may also be more sensitive to valence-specific differentiation compared to the early-window LPP [59]. Consequently, individuals with obesity and IR would be expected to have higher late-window LPP amplitudes and enhanced affective processing in response to unpleasant stimuli in conjunction with lower late-window LPP amplitudes and attenuated affective processing in response to pleasant stimuli compared to lean and insulin-sensitive individuals, respectively.

This study aimed to investigate whether obesity and/or IR moderate the links between brain potentials measured via EEG and underlying negative and/or positive affective processing in younger adults. The central hypotheses were: (1) younger adults without obesity or IR will show attenuated negative affective processing through lower late-window LPP amplitudes in response to unpleasant visual stimuli compared to respective counterparts; and (2) young adults without obesity or IR will show enhanced positive affective processing through higher late-window LPP amplitudes in response to pleasant stimuli compared to respective counterparts.

## 2. Materials and Methods

### 2.1. Participants

A power analysis was conducted utilizing G*Power (Version 3.1.9.7; The G*Power Team, Düsseldorf, Germany [60,61]) to determine the appropriate sample size to test whether body fat percentage (BF%) and/or IR values modify the correlations between brain potentials and affective processing parameters using hierarchical moderated regression analysis. An a priori *F*-test power analysis determined that a sample size of 42 participants was required to test a linear multiple regression, fixed model, *R*^2^ increase, with a large effect size (Cohen’s *f*^2^ = 0.35, which is commonly reported in LPP research (e.g., [44,62])), *α* = 0.0063 (Bonferroni corrected), power = 0.80, 1 tested predictor, and 7 total predictors (3 main predictors, 4 covariates). However, considering that attrition rates across all clinical studies average 30% [63], the sample size was adjusted according to the following formula, as previously recommended [64]: 42/(1 − 0.30). Based on this formula, 60 participants were required to adjust for anticipated attrition rates.

Although 60 younger adults between 18–39 years of age who inhabited the Ames, Iowa, USA location were invited to participate, higher-than-anticipated attrition (*n* = 24) and missing (*n* = 5) or excessively noisy (*n* = 1) EEG data resulted in the inclusion of 30 participants in the final analyses. Consequently, statistical power was evaluated using G*Power (Version 3.1.9.7; The G*Power Team, Düsseldorf, Germany [60,61]) for each moderation model that was found to have a significant interaction term; these power analyses are described alongside each significant model in Section 3. In addition, to ensure no selection bias was present between the participants who were included in versus excluded from the final analyses, key participant characteristics were compared (see Supplementary Table S1). No differences were detected in any of the key characteristics between participants who were included versus excluded from the final analyses, confirming that no selection bias was present and missingness was random.

Inclusion criteria were:

1. aged 18–39 years; and
2. overnight fasting capability (≤16 consecutive hours).

Exclusion criteria were:

1. cerebrovascular/neurological diseases (e.g., stroke, multiple sclerosis, traumatic brain injury, cerebral hematoma);
2. major cardiovascular events;
3. major psychiatric disorders (e.g., major depressive disorder, generalized anxiety disorder, schizophrenia, autism spectrum disorders, attention deficit hyperactivity disorder, bipolar disorder);
4. recent drug, alcohol, or substance abuse (≤6 months);
5. diabetes mellitus I/II;
6. blood pressure ≥ 21.3/14.7 kPa (≥160/110 mmHg);
7. fasting blood glucose values of <3.9 mmol/L (<70 mg/dL) or >6.9 mmol/L (>125 mg/dL);
8. current use of medications that impact weight, insulin levels, serum biomarkers, or affective processing (e.g., systemic corticosteroids, weight reduction medications, atypical antipsychotics);
9. ferrous metal implants or shrapnel around the head/eyes;
10. currently pregnant; and/or
11. currently using nicotine/tobacco products.

Approval from the Iowa State University Institutional Review Board was obtained.

### 2.2. Study Design and Protocol

A cross-sectional, observational design was utilized to test if obesity and/or IR moderate brain potentials and affective processing outcomes. Study visits included an initial screening and two follow-up visits within a one-month timeframe.

#### 2.2.1. Initial Screening Visit

Each potential participant had the consent form thoroughly explained, with signatures obtained during the initial screening visit prior to commencing any study procedures. Subsequently, the medical history of each potential subject was thoroughly queried. Additionally, height and weight were obtained.

#### 2.2.2. First Follow-Up Visit

During the first follow-up visit, height and weight were reobtained, and each participant’s BF% was measured by a dual-energy X-ray absorptiometry scan (DXA; Hologic Discovery Version 12.3; Hologic, Inc., Marlborough, MA, USA). Fasting blood serum was collected, from which fasting glucose and insulin values were quantified. Utilizing these values, IR was gauged for each participant in accordance with the following Homeostatic Model Assessment for Insulin Resistance (HOMA-IR) formula: (fasting glucose (mmol/L) × fasting insulin (pmol/L))/135 (or (glucose (mg/dL) × insulin (µIU/mL))/405 for conventional units) [65]. Physical activity levels were self-reported as part of the body composition analysis and categorized as: (1) sedentary (no engagement in physical activity beyond basic movements from daily life); (2) low active (<150 (moderate) or <75 (vigorous) minutes/week); (3) active (150–300 (moderate) or 75–150 (vigorous) minutes/week); or (4) very active (>300 (moderate) or >150 (vigorous) minutes/week [66]). Participants were subsequently dichotomized by whether physical activity levels totaled <150 (moderate) or <75 (vigorous) minutes weekly (sedentary/low active) versus ≥150 (moderate) or ≥75 (vigorous) minutes weekly (active/very active), as done previously [67]. Specifically, for these analyses, self-reported physical activity levels were dummy-coded as a binary variable, with 0 representing weekly physical activity levels of ≥150 (moderate) or ≥75 (vigorous) minutes (i.e., active to very active) and 1 representing weekly physical activity levels of <150 (moderate) or <75 (vigorous) minutes (i.e., sedentary to low active). The physical activity dummy-coded variable was then included as a covariate in each step for each hierarchical moderated model tested, along with all other covariates (i.e., age (years), sex (male/female), and racial/ethnic group (Whites, Asians, Blacks, and Hispanics/Latinxs)).

#### 2.2.3. Second Follow-Up Visit

During the second follow-up visit, subjects passively viewed pictures during an International Affective Picture System (IAPS) task [68], which was adapted from procedures described previously (see Supplementary Figure S1 [69,70]). Simultaneously, neural activity was recorded via EEG. Specifically, participants were singly shown 64 randomized images with normative arousal ratings [68,71]—including neutral (*n* = 21; arousal = 2.96 ± 0.83), unpleasant (*n* = 23; arousal = 6.16 ± 0.75), and pleasant (*n* = 20; arousal = 4.53 ± 1.84) images—for one second each. Before viewing each image, subjects were shown one of three possible visual cues for two seconds each, which included “X” (signifying an upcoming emotional visual stimulus), “O” (indicating an upcoming neutral visual stimulus), or “?” (no information about upcoming image content). After the cue presentation, a jittered inter-stimulus interval (ISI) of between 2–8 s was followed by the presentation of each image for 1 s in a randomized valence order. Subsequently, an additional jittered ISI of 5–9 s was presented. Thereafter, participants were then given four seconds to rate the magnitude of pleasantness or unpleasantness of each preceding image (i.e., valence ratings), where 1 = “very positive,” 2 = “somewhat positive,” 3 = “somewhat negative,” and 4 = “very negative.” The task photo identification numbers used in this study are listed in Table A1. IAPS task parameters were scored in E-Prime*3 [72].

Affective processing parameters included the mean valence ratings (an indicator of the magnitude of negativity or positivity felt in response to unpleasant or pleasant visual stimuli, respectively [73]) and stimulus-to-response-onset reaction times (RTs, which reflect affective processing and attentional engagement [74] and are sensitive to valence-specific differentiation [75]) to unpleasant, pleasant, and neutral images. As done in prior work [76,77,78,79], in order to optimally gauge valence-specific affective processing, the scores of each emotionally valenced condition (i.e., Negative, Positive) were subsequently compared to the scores of the control (i.e., Neutrally valenced) or oppositely valenced conditions as follows: (1) Negative minus Neutral; (2) Positive minus Neutral; and (3) Negative minus Positive. However, because valence ratings reflect opposite degrees of unpleasantness or pleasantness for emotionally valenced pictures, the calculation for the mean valence rating scores in the oppositely valenced contrasted condition (i.e., Negative minus Positive) was accordingly adjusted as: Negative minus (4 − Positive). For the contrasted mean valence rating parameters, larger positive difference scores (i.e., higher numerical values) in the contrasted Negative−Neutral and Negative−Positive conditions indicated a greater magnitude of unpleasantness given via subjective ratings to the visual stimuli overall, whereas less negative difference scores (i.e., higher numerical values) in the contrasted Positive−Neutral condition reflected a greater magnitude of pleasantness given via subjective ratings to the visual stimuli overall. For the contrasted mean stimulus-to-response-onset RT parameters, difference scores with negative values represent faster RTs to the emotional condition versus the Neutrally or oppositely valenced condition, whereas difference scores with positive values instead reflect faster RTs to the Neutrally or oppositely valenced condition. 

### 2.3. Electroencephalography

As described previously [67], EEG was recorded using an electrode array cap comprising 64 Ag/AgCl electrodes (Sands Research, El Paso, TX, USA), in alignment with the standards defined by the International 10–10 System of electrode placement, and was interfaced to a digital biopotential amplifier (DBPA-1; Sensorium Inc., Charlotte, VT, USA). A sampling rate of 2048 Hz was used. Utilizing EEGLAB (Version 2021.1; [80]) in MATLAB (Version R2022a; MathWorks, Inc., Natick, MA, USA), the EEG preprocessing procedure included:

1. application of a high-pass (≤0.1 Hz) filter followed by a low-pass (≥30 Hz) filter (using gain 1000, 16-bit A/D conversion). The utilization of a 0.1–30 Hz bandpass has been the most frequent recommendation for the majority of cognitive and affective studies in prior work [81]. These bandpass filters are furthermore in alignment with recent methodological papers that tested the optimal filter settings for slow-evolving components [82] and the optimal pre-processing parameters for LPP quantification specifically [83];
2. considering that the LPP component persists for approximately 1000 ms post-image presentation [84], continuous EEG data were epoched into two-second segments post-stimulus onset (0–2.0 s latency window);
3. whole-brain amplitude values were standardized via average reference computation;
4. eliminated noisy channels through an automatic epoch rejection method (channel fluctuations ≥ 1000 µV);
5. computed an Independent Component Analysis (ICA) using the Multiple Artifact Rejection Algorithm (MARA) toolbox (Version 1.2; [85,86]);
6. eliminated artifacts using a peer-reviewed algorithm and statistical parameters [87]; and
7. exported the whole time-course grand means for each valence condition to a .csv file.

### 2.4. Event-Related Potential Components

Grand-mean averaged waveforms were utilized to quantify the EPN and LPP components as follows:

1. EPN: six occipitotemporal electrodes (O1/O2, PO7/PO8, P7/P8; [88,89]) between 200–280 ms post-stimulus exposure [90,91]; and
2. LPP: ten centroparietal electrodes (CPz, CP1/CP2, CP3/CP4, Pz, P1/P2, P3/P4; [92,93]) across three latency windows, defined as the early (400–800 ms), middle (800–1200 ms), and late (1200–2000 ms) windows [94,95].

To serve as ERP indices of valence-specific affective processing, EPN and LPP amplitudes of each emotionally valenced condition (i.e., negative, positive) were then compared to the amplitudes of the control (i.e., Neutrally valenced) or oppositely valenced conditions as follows [76,77,78,79]:

1. Negative−Neutral;
2. Positive−Neutral; and
3. Negative−Positive.

For the EPN component, lower (i.e., more negative) difference score values reflected overall larger amplitude deflections in response to the emotionally valenced condition compared to the Neutrally or oppositely valenced condition. For the LPP component, higher (i.e., more positive) difference scores indicated overall larger amplitude deflections in response to the emotionally valenced condition compared to the Neutrally or oppositely valenced condition.

### 2.5. Statistical Analysis

Participants were stratified into two separate groups based on BF% (lean (*n* = 8) versus obese (*n* = 22)) and HOMA-IR (insulin-sensitive (*n* = 18) versus insulin-resistant (*n* = 12)) values to assess between-group differences. Subjects without and with obesity were respectively defined as having a BF% of <25% (males) or <35% (females) versus ≥25% (males) or ≥35% (females) [96]. As previously recommended [97,98], HOMA-IR values of <2.0 versus ≥2.0 defined insulin sensitivity versus IR, respectively. Statistical comparisons of participant characteristics were conducted using chi-square, Fisher’s exact, or independent-samples *t*-tests in SPSS Statistics (Version 31.0.0.0; IBM Corp., Armonk, NY, USA). Significance was set at *p* ≤ 0.05 for group comparisons.

Hierarchical moderated regression analyses tested whether obesity and/or IR moderated the relationships between brain potentials and affective processing utilizing the PROCESS macro for SPSS (Version 5.0; [99]). All continuous variables (i.e., EPN and LPP amplitudes, valence ratings, RTs, BF% values, HOMA-IR values, age) were first assessed for frequency distributions and skewness/kurtosis. Nonparametric continuous variables were square root-transformed or logarithmic-transformed as necessary, with sensitivity analyses conducted utilizing the original and transformed variables to evaluate differences in model statistics [100]. Outliers were assessed via the standardized residual (≥2.5) and Mahalanobis distance criteria [101], and all detected outliers were Winsorized via replacement with values at the 5th and 95th percentiles for the lowest and highest values, respectively [102]. For mean valence ratings across all independent and contrasted valence conditions, ceiling and floor effects were evaluated by calculating the proportions of subjects who self-reported mean valence ratings that corresponded to the minimum (1) and maximum (4) valence ratings possible. Furthermore, all continuous variables were mean-centered prior to analysis to eliminate multicollinearity issues. A multiple imputation procedure in SPSS was applied to impute missing affective processing parameters (≤10%). The first step of each moderation model tested whether the predictor and moderator variables independently accounted for a significant proportion of the variance for the outcome variable. For each contrasted valence condition, the first step included a predictor (EPN, LPP voltage amplitudes), a moderator (BF%, HOMA-IR), an outcome variable (valence ratings, RTs), and all significant (*p* ≤ 0.10) covariates (age, physical activity, racial/ethnic group, sex). In the second step, each moderation model was retested after the addition of the interaction term (predictor × moderator), with all covariates readded. In both steps, each non-significant covariate was singly eliminated from each model in descending order of significance. The Bonferroni correction was applied to correct for multiple comparisons of associations, where *p* ≤ 0.0063 was considered statistically significant.

## 3. Results

### 3.1. Participant Characteristics

As delineated in Table 1, demographic and clinical characteristics were compared between stratified adiposity (lean versus obese) and IR (insulin-sensitive versus insulin-resistant) groups. In summary, subjects with obesity and IR had more insulin dysregulation compared to respective counterparts. Additionally, subjects with obesity showed greater glucose dysregulation. Finally, subjects with IR participated in less weekly physical activity and had higher BF% values and diastolic blood pressure.

### 3.2. Event-Related Potentials

**Figure 1:**
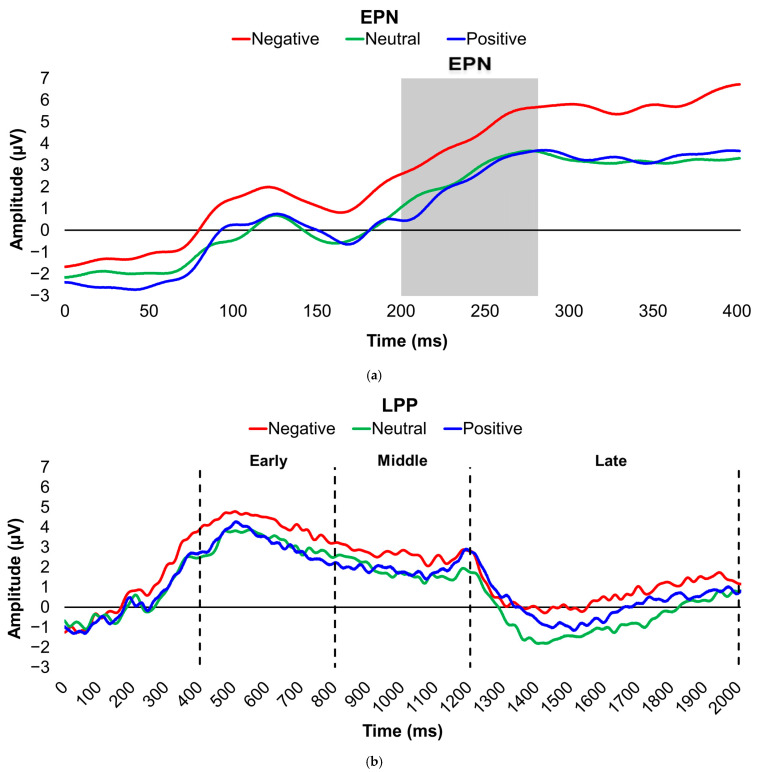
Line graphs depict the grand-mean averaged EPN (**a**) and LPP (**b**) waveforms across all participants after viewing Negatively (red), Neutrally (green), and Positively valenced (blue) pictures during an International Affective Picture System task. In the EPN line graph, the gray box denotes the post-stimulus period of interest for the EPN component (200–280 ms). In the LPP line graph, the vertical, dashed lines denote the post-stimulus periods of interest for the early (400–800 ms), middle (800–1200 ms), and late (1200–2000 ms) latency windows of the LPP component. Amplitude was measured in microvolts (µV); time was measured in milliseconds. EPN = early posterior negativity; LPP = late positive potential; ms = milliseconds.

EPN and LPP grand-mean averaged waveforms across the sample are illustrated for each valence condition in Figure 1a,b. For EPN (Figure 1a), Positively valenced images elicited the largest (i.e., most negative) amplitudes, while Negatively valenced images evoked the smallest. For LPP (Figure 1b), Negatively valenced pictures generated the largest (i.e., most positive) amplitudes across all latency windows, and Neutrally valenced pictures evoked the smallest during the middle and late latency windows.

### 3.3. Neural Amplitudes by Categorical Groups

**Figure 2:**
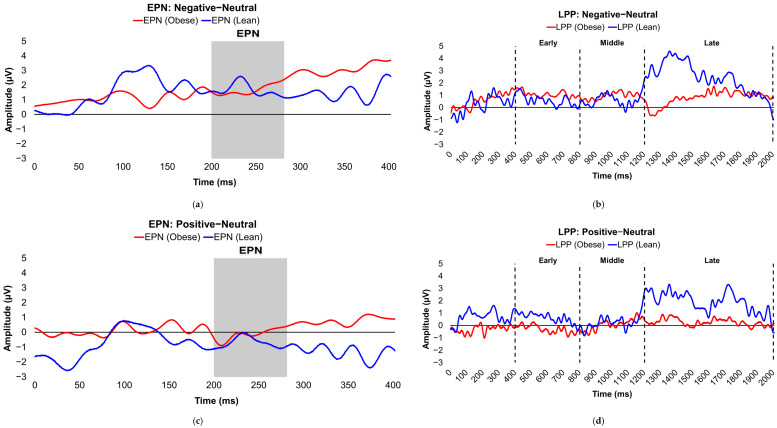

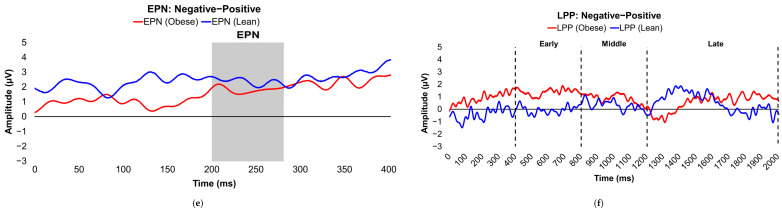
Line graphs show the comparisons of the differenced grand-mean averaged EPN (left) and LPP (right) waveforms in the contrasted picture conditions (i.e., Negative minus Neutral (**a**,**b**), Positive minus Neutral (**c**,**d**), and Negative minus Positive (**e**,**f**) EPN and LPP waveforms) between lean (body fat percentage of <25% (males) and <35% (females), in blue) versus obese (body fat percentage of ≥25% (males) and ≥35% (females), in red) subjects. For the EPN component, lower (i.e., more negative) difference score values reflect overall larger amplitude deflections in response to the emotionally valenced condition compared to the Neutrally or oppositely valenced condition. For the LPP component, higher (i.e., more positive) difference scores indicate overall larger amplitude deflections in response to the emotionally valenced condition compared to the Neutrally or oppositely valenced condition. In the EPN line graphs, the gray box denotes the post-stimulus period of interest for the EPN component (200–280 ms). In the LPP line graphs, the vertical, dashed lines denote the post-stimulus periods of interest for the early (400–800 ms), middle (800–1200 ms), and late (1200–2000 ms) latency windows of the LPP component. Amplitude was measured in microvolts (µV); time was measured in milliseconds. EPN = early posterior negativity; LPP = late positive potential; ms = milliseconds.

**Figure 3:**
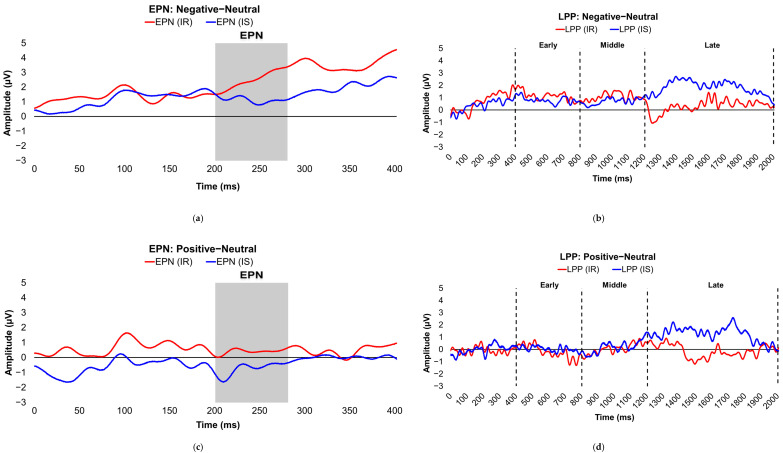

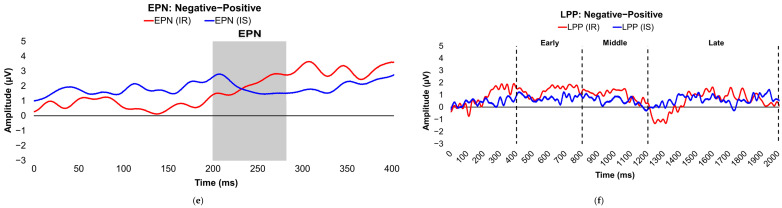
Line graphs show the comparisons of the differenced grand-mean averaged EPN (left) and LPP (right) waveforms in the contrasted picture conditions (i.e., Negative minus Neutral (**a**,**b**), Positive minus Neutral (**c**,**d**), and Negative minus Positive (**e**,**f**) EPN and LPP waveforms) between insulin-sensitive (HOMA-IR values of <2.0, in blue) versus insulin-resistant (HOMA-IR values of ≥2.0, in red) subjects. For the EPN component, lower (i.e., more negative) difference score values reflect overall larger amplitude deflections in response to the emotionally valenced condition compared to the Neutrally or oppositely valenced condition. For the LPP component, higher (i.e., more positive) difference scores indicate overall larger amplitude deflections in response to the emotionally valenced condition compared to the Neutrally or oppositely valenced condition. In the EPN line graphs, the gray box denotes the post-stimulus period of interest for the EPN component (200–280 ms). In the LPP line graphs, the vertical, dashed lines denote the post-stimulus periods of interest for the early (400–800 ms), middle (800–1200 ms), and late (1200–2000 ms) latency windows of the LPP component. Amplitude was measured in microvolts (µV); time was measured in milliseconds. EPN = early posterior negativity; IR = insulin-resistant; IS = insulin-sensitive; LPP = late positive potential; ms = milliseconds.

Table 2 presents the comparisons of the differenced grand-mean averaged EPN and LPP waveforms in each contrasted picture condition (i.e., Negative minus Neutral, Positive minus Neutral, and Negative minus Positive) by stratified adiposity (also see Figure 2a–f (differenced waveforms) and Supplementary Figure S2a–f (non-differenced waveforms for each separate valence condition)) and IR levels (also see Figure 3a–f (differenced waveforms) and Supplementary Figure S3a–f (non-differenced waveforms for each separate valence condition)). Concisely, participants without obesity or IR exhibited higher late-window LPP amplitudes to unpleasant versus neutral pictures compared with lean or insulin-sensitive counterparts, respectively (see Figure 2b/Supplementary Figure S2b (lean versus obese participants) and Figure 3b/Supplementary Figure S3b (insulin-sensitive versus insulin-resistant participants)). Furthermore, participants with obesity evoked larger LPP amplitudes during the early latency window in the contrasted Negative−Positive valence condition (see Figure 2f/Supplementary Figure S2f). ERPs were also compared between stratified obesity and insulin groups by separate valence conditions in Supplementary Table S2, which showed that insulin-sensitive subjects had significantly higher late-window LPP amplitude deflections in response to unpleasant images than did subjects with IR.

### 3.4. Affective Processing Parameters by Categorical Groups

Differences in affective processing parameters were assessed by stratified adiposity and IR groups for each contrasted valence condition (see Table 3) and for each separate valence condition (see Supplementary Table S3). In summary, participants with IR had slower RTs in response to unpleasant versus neutral images compared to insulin-sensitive subjects. No significant differences in affective processing parameters were detected between participants with and without obesity.

### 3.5. Neural Amplitudes and Affective Processing

The correlation matrices between the ERPs and affective processing parameters are displayed for each contrasted valence condition in Table 4 and for each separate valence condition in Supplementary Table S4. Succinctly, neither EPN nor LPP amplitudes were found to directly correlate with any of the affective processing parameters across any of the contrasted valence conditions, whereas EPN and early-window LPP amplitudes positively associated with valence ratings in the separate Negatively valenced condition.

### 3.6. Moderation Analyses

#### 3.6.1. Negative−Neutral Picture Condition

**Figure 4:**
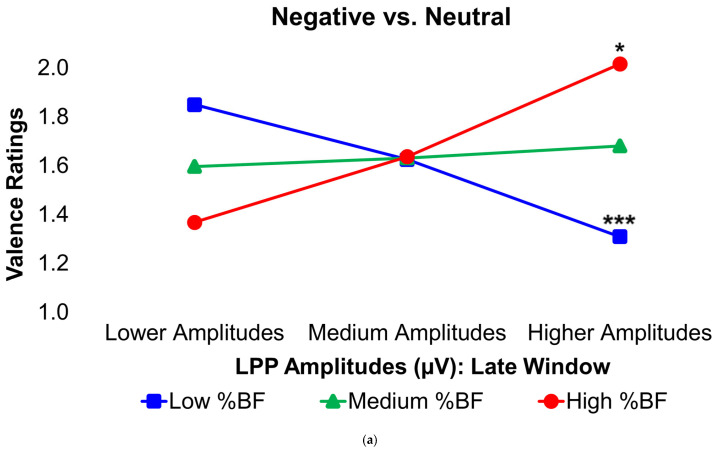

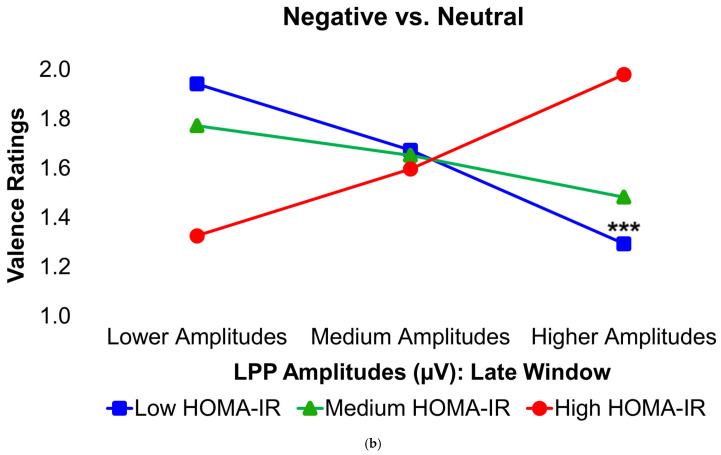
Interaction plots depict how body fat percentage (**a**) and HOMA-IR values (**b**) moderated the relationships between the voltage amplitudes of the LPP component during the late latency window and mean valence ratings in the contrasted Negative−Neutral picture condition. Predictor (late-window LPP voltage amplitudes) and moderator (BF%, HOMA-IR) variables were tested as continuous variables but are shown as tertiles for visualization. Amplitude deflections (lower, medium, higher) and BF% or HOMA-IR levels (low, medium, high) respectively reflect values at the 16th, 50th, and 84th percentiles of the total sample. Valence ratings represent the mean ratings of the magnitude of pleasantness or unpleasantness of images for each valence condition, where 1 = “very positive,” 2 = “somewhat positive,” 3 = “somewhat negative,” and 4 = “very negative,” which were subsequently contrasted as the difference between the mean valence ratings of Neutrally valenced pictures from the mean valence ratings of Negatively valenced pictures (i.e., Negative minus Neutral valence ratings). For the LPP component, higher (i.e., more positive) difference scores indicate overall larger amplitude deflections in response to unpleasant pictures compared to neutral pictures. For the contrasted mean valence rating parameter, larger (i.e., more positive) difference scores in the contrasted Negative−Neutral condition indicate a greater magnitude of unpleasantness given via subjective ratings to the visual stimuli overall. For subjects with low BF% and HOMA-IR values, greater (i.e., more positive) LPP amplitudes during the late latency window were linked to less negative valence ratings in response to unpleasant versus neutral pictures. In contrast, the reverse pattern was found in those with high BF%, and no significant association was found in those with high HOMA-IR values. BF% = body fat percentage; HOMA-IR = homeostatic model assessment for insulin resistance; LPP = late positive potential; ms = milliseconds. * and *** = *p*’s ≤ 0.05 and ≤0.005, respectively.

The results of how BF% and HOMA-IR values moderated the relationships between neural activity and affective processing scores in response to unpleasant versus neutral pictures are shown in Table 5 for valence ratings and in Table 6 for RTs. Further slope analyses in Figure 4a,b illustrate that, in participants with low BF% and HOMA-IR values, greater LPP amplitudes during the late latency window were associated with less negative valence ratings in response to unpleasant pictures relative to neutral pictures. In contrast, the opposite relationship was true in those with high BF%, where larger late-window LPP amplitudes were correlated with more negative valence ratings in response to unpleasant versus neutral images (Figure 4a). A similar trend was also observed in those with high HOMA-IR levels, although this did not reach statistical significance (Figure 4b).

Considering the smaller sample size, two post hoc power analyses were conducted to evaluate the statistical power of both models with significant interactions. Utilizing G*Power (Version 3.1.9.7; The G*Power Team, Düsseldorf, Germany [60,61]), a post hoc *F*-test was conducted using a linear multiple regression, fixed model, *R*^2^ increase statistical approach, with *N* = 30, calculated effect sizes of Cohen’s f*^2^* = 0.45 (for the late LPP × BF% interaction) and 0.35 (for the late LPP × HOMA-IR interaction), *α* = 0.0063 (Bonferroni corrected *p*-value), and 1 tested predictor, and 3 total predictors (3 main predictors, no significant covariates), the statistical power was determined to be 75.1% for the late LPP × BF% interaction model and 60.9% for the late LPP × HOMA-IR interaction model when predicting mean valence ratings in the contrasted Negative−Neutral valence condition. Although this statistical power is modest and does not meet the standard 80% power threshold, these results nonetheless provide meaningful preliminary evidence. The observed large effect sizes in the interaction models (*f*^2^ ≈ 0.35–0.45) suggest that the detected associations are unlikely to be artifacts of insufficient power alone. Thus, while these findings should be interpreted with appropriate caution, these preliminary results nonetheless remain informative and warrant further investigation in larger samples.

#### 3.6.2. Positive−Neutral Picture Condition

Table 7 and Table 8 present the results of the moderation analyses for predicting valence ratings and RTs, respectively, in the Positive−Neutral trials. Contrary to what was anticipated, BF% and IR were not found to moderate the links between EPN or LPP amplitudes and affective processing scores for pleasant versus neutral stimuli.

#### 3.6.3. Negative−Positive Picture Condition

Results of the moderation analyses are displayed in Table 9 for valence ratings and Table 10 for RTs in the Negative−Positive condition. In contrast with what was hypothesized, BF% and IR did not moderate the links between EPN or LPP amplitudes and affective processing parameters for unpleasant stimuli relative to pleasant.

## 4. Discussion

Evidence to support whether obesity is associated with deficits in emotional processing in young adults has been inconsistent in the prevailing literature. Furthermore, whether IR is linked to affective processing impairments in otherwise healthy young adults remains unexplored to date. Moreover, neuroelectrophysiological investigations of whether adiposity and IR modify the correlations between the temporal and electrophysiological aspects of neural activity and affective processing in young adults are lacking. Therefore, EEG was utilized to investigate whether obesity and/or IR moderated brain potentials and associated affective processing outcomes in young adults.

In the Negative−Neutral trials, young adults without obesity or IR displayed significantly greater LPP amplitudes during the late latency window compared to young adults with obesity or IR, respectively, in contrast with what was expected. Moreover, in young adults without obesity or IR, larger LPP responses were linked to less negative ratings of unpleasant versus neutral images, suggesting that stronger neural engagement was associated with a dampening of negative emotional experience. By contrast, this relationship was reversed in young adults with obesity, indicating a disruption in the typical coupling between neural activity and emotional evaluation in these individuals. Ordinarily, increased activity in the dlPFC in response to unpleasant stimuli reflects more top-down control to suppress affective responses [22], with successful down-regulation of Negatively valenced stimuli reflected by smaller LPP responses to unpleasant stimuli [103]. Other evidence has suggested that higher late latency window LPP amplitudes could also indicate more difficulties with diverting attention from unpleasant stimuli [104]. Hence, the association between higher late-window LPP amplitude responses and less negative emotional rating responses to unpleasant stimuli was unexpected for lean, insulin-sensitive young adults. Despite this, the inverse LPP amplitudes–valence ratings correlation found in this study is in congruence with findings from several earlier studies (e.g., [105,106,107,108]). Such findings could imply that, rather than indicating an unsuccessful dampening of affective responses and/or greater difficulties with attention diversion from Negatively valenced stimuli, higher LPP amplitude deflections during the late latency window may instead reflect more effortful spontaneous emotion regulation, as previously suggested [105,108]. Taken together, these findings could suggest that lean, insulin-sensitive individuals may attenuate negative affective processing of unpleasant stimuli through greater cognitive effort in order to automatically regulate Negatively valenced visual stimuli spontaneously during passive picture-viewing tasks. However, it should be noted that current evidence to support this hypothesis remains scarce and, therefore, warrants further investigation.

While these results show that higher BF% and IR weaken the associations between brain potentials and negative affective processing in young adults, prior work has suggested that IR may be one mechanism through which obesity alters the neural basis of negative affective processing rather than acting as isolated moderators. Excess body fat has been identified as an independent inducer of IR [109,110,111], predominantly via the promotion of cell dysfunction and inflammation [112]. Given this, it is unsurprising that obesity and IR are risk factors for many of the same health conditions, including mood disorders [2,3,19,30] and emotional processing modifications [7,9,18]. Although this is a feasible hypothesis, this study lacked the statistical power to test both moderators simultaneously. Therefore, future research should test these associations utilizing a larger sample and longitudinal designs.

In the contrasted Positive−Neutral valence trial, neither obesity nor IR were found to moderate the links between LPP amplitudes across any of the latency windows and affective processing scores, implying that neural responses to pleasant stimuli and associated positive affective processing may be spared from obesity- and/or IR-induced brain structural and functional alterations in young adulthood. These results are in concurrence with some [104,113], but not all [114,115], previous findings. The null results found for positive affective processing parameters in this study may be in part explained by differences in neurocircuitry between negative and positive affective processing. Although negative and positive affective processing share overlapping neurocircuitry, some distinctions have been identified in prior work. Specifically, the processing of negative emotions more heavily involves activity from the amygdala, aINS, ACC, visual cortex, dlPFC, and vlPFC [116,117], whereas the processing of positive emotions more substantially relies upon activity in the medial PFC, OFC, ventral striatum, nucleus accumbens, and ventral tegmental area [118,119]. These findings could suggest that brain structures that play more significant roles in processing information associated with pleasant stimuli may be less affected by obesity- and/or IR-linked brain structural and functional alterations compared to structures that have more involvement in modulating negative affective processing. This is in alignment with evidence showing that excessive adiposity and metabolic disturbances disproportionately affect the amygdala–insula–cingulate circuits, dlPFC, and vlPFC [17,120,121,122,123], while mesolimbic structures that are more functionally involved in positive affective processing show less consistent results [24,30,124,125,126], especially in young adults [124,125,126]. Still, future studies will be crucial to clarify whether obesity and IR impact brain potentials and positive affective processing in young adulthood.

Moreover, obesity and IR did not moderate the relationships between brain potentials and underlying affective processing in the contrasted Negative−Positive condition. This suggests that, similarly to early-window LPPs, late-window LPPs may also be more strongly driven by arousal than by valence and, thus, may not be valence-biased [48,127,128], although this contrasts some prior study findings (e.g., [59]). Moreover, inconsistencies exist in the body of literature that has compared the LPP responses to unpleasant versus pleasant stimuli, with some studies finding no differences in LPP amplitudes between either emotional condition across a wide range of age groups [50,129] and other studies finding valence-specific differentiation, although these findings appeared to be arousal-dependent [59,128]. Significant methodological, LPP quantification, and sample heterogeneity may partially account for such discordant findings.

Finally, BF% and HOMA-IR did not moderate the links between EPN amplitudes and affective processing parameters across any of the contrasted valence conditions. Hence, this suggests that obesity and IR do not impact visual attention allocation to emotional versus neutral stimuli in young adulthood. As far as the authors are aware, this study is the first to investigate whether obesity and/or IR moderated the relationship between EPN amplitudes and affective processing. Nonetheless, these results are not surprising when considering that the scalp distribution of the EPN component over the occipitotemporal sites reflects visual cortical activity [130], a largely insulin-independent region [131]. A study demonstrated this by showing that insulin infusion had no effect on subsequent visual evoked potentials [132]. Nevertheless, additional research is needed to clarify whether obesity and/or IR influence EPN amplitudes and underlying visual attention allocation to emotional versus neutral stimuli.

This study was not without limitations. First, the sample size was small, hence limiting the generalizability of this study’s findings and barring the simultaneous testing of both moderators in one model due to being underpowered. Second, the utilization of the EEG neuroimaging modality in conjunction with the cross-sectional study design barred any causal implications. Third, neural activity measured during exposure to emotional images in a laboratory setting may not necessarily translate to real-world settings. Fourth, HOMA-IR is an index of short-term IR and may not have accurately captured chronic IR in all subjects.

This study also included several strengths. First, the central research questions were novel. Second, EEG provided an inexpensive, non-invasive neuroimaging approach with superior temporal resolution compared to fMRI [33,34,35]. Third, body composition was measured directly via DXA, which provided a significantly more accurate quantification of BF% in comparison to indirect measures (e.g., BMI [133]).

## 5. Conclusions

This study provides preliminary evidence that obesity and IR moderate the relationship between late-stage brain potentials and negative affective processing, but not positive affective processing, in young adults. These data furthermore support some prior findings that obesity and IR are both independent risk factors for deficits in affective processing in young adulthood. Most notably, lean and insulin-sensitive individuals showed attenuated negative affective processing when late-window LPP amplitudes were larger, whereas this pattern was reversed or absent among those with greater adiposity or IR. These preliminary findings suggest that higher body fat levels and IR may disrupt the typical coupling between neural activity and emotional evaluation of unpleasant stimuli during young adulthood. These events may, in turn, increase susceptibility to mood disorders among this age group. Young adulthood is when mood disorders most commonly manifest; hence, altered late-stage affective processing may reflect an early neurobiological vulnerability that precedes overt symptoms. If obesity and IR contribute to inefficient neural regulation of negative emotion, this could imply that these factors may accelerate trajectories toward heightened negative affectivity or later mood dysregulation. The identification of such patterns in metabolically at-risk young adults could inform early screening approaches and highlight opportunities for interventions, including lifestyle modification, which may, in turn, strengthen affective regulation before clinical symptoms develop. Additional work is warranted to investigate these interrelationships longitudinally with larger sample sizes of young adults using EEG, particularly for positive affective processing.

## Figures and Tables

**Table 1:** Participant characteristic comparisons by adiposity and insulin groups.

		Adiposity			Insulin		
Data (Unit)	Total (*N* = 30)	Lean (*n* = 8)	Obese (*n* = 22)	*t*-Value/chi-Square	Insulin-Sensitive (*n* = 18)	Insulin-Resistant (*n* = 12)	*t*-Value/chi-Square
Age (years)	25.7 (5.3)	23.3 (4.1)	26.5 (5.5)	*t* = −1.54	24.9 (5.6)	26.8 (4.9)	*t* = −0.98
Sex (*n* females (%))	15 (50.0%)	3 (37.5%)	12 (54.5%)	*χ*^2^ = 0.68	8 (44.4%)	7 (58.3%)	*χ*^2^ = 0.56
**Activity Level (** ***n*** ** (%))**				*χ*^2^ = 4.18			***χ*** ^2^ = 5.63 *
***Sedentary/Low Active***	20 (66.7%)	3 (37.5%)	17 (77.3%)		**9 (50.0%)**	**11 (91.7%)**	
***Active/Very Active***	10 (33.3%)	5 (62.5%)	5 (22.7%)		**9 (50.0%)**	**1 (8.3%)**	
Race/Ethnicity (*n* (%))				*χ*^2^ = 3.62			*χ*^2^ = 5.17
*White*	24 (80.0%)	5 (62.5%)	19 (86.4%)		13 (72.2%)	11 (91.7%)	
*Asian*	5 (16.7%)	3 (37.5%)	2 (9.1%)		5 (27.8%)	0 (0.0%)	
*Hispanic/Latinx*	1 (3.3%)	0 (0.0%)	1 (4.5%)		0 (0.0%)	1 (8.3%)	
*Black*	0 (0.0%)	0 (0.0%)	0 (0.0%)		0 (0.0%)	0 (0.0%)	
**BF% (DXA)**	37.1 (10.3)	**24.8 (4.7)**	**41.6 (7.9)**	***t *** = −5.64 ***	**31.6 (8.5)**	**45.5 (6.7)**	***t *** = −4.76 ***
**BMI**	29.3 (8.2)	**21.0 (1.6)**	**32.2 (7.5)**	***t *** = −6.94 ***	**24.5 (5.3)**	**36.4 (6.4)**	***t *** = −5.74 ***
**BP, diastolic (kPa)**	10.2 (1.7)	9.6 (2.0)	10.5 (1.5)	*t* = −1.26	**9.7 (1.6)**	**11.1 (1.4)**	***t *** = −2.33 *
BP, systolic (kPa)	16.2 (2.1)	16.6 (2.8)	16.0 (1.7)	*t* = 0.68	16.0 (2.4)	16.6 (1.3)	*t* = −0.75
**Glucose (mmol/L), fasting**	5.0 (0.5)	**4.7 (0.4)**	**5.1 (0.4)**	***t *** = −2.38 *	4.9 (0.5)	5.2 (0.5)	*t* = −1.50
Height (meters)	1.7 (0.1)	1.8 (0.1)	1.7 (0.1)	*t* = 0.43	1.7 (0.1)	1.7 (0.1)	*t* = −0.37
Hemoglobin A1c (%)	5.4 (0.2)	5.3 (0.1)	5.4 (0.2)	*t* = −1.05	5.3 (0.2)	5.4 (0.2)	*t* = −1.36
**HOMA-IR**	2.6 (2.5)	**0.9 (0.3)**	**3.3 (2.7)**	***t *** = −4.24 ***	**1.2 (0.4)**	**4.9 (2.8)**	***t *** = −8.07 ***
**Insulin (pmol/L), fasting**	69.3 (63.0)	**25.2 (8.5)**	**85.4 (66.7)**	***t *** = −4.14 ***	**31.8 (9.5)**	**125.6 (67.5)**	***t *** = −4.78 ***

Values are Mean (Standard Deviation) unless otherwise indicated. For the stratified adiposity groups, *t*-values, chi-square, and Fisher’s exact values reflect differences between participants with and without obesity based on a body fat percentage of ≥25% (males) and ≥35% (females) versus <25% (males) and <35% (females), respectively. For the stratified insulin groups, *t*-values, chi-square, and Fisher’s exact values reflect differences between participants with insulin resistance and insulin sensitivity based on HOMA-IR values of ≥2.0 versus <2.0, respectively. BF% = body fat percentage; BMI = body mass index; BP = Blood pressure; DXA = dual-energy X-ray absorptiometry; hemoglobin A1c = glycated hemoglobin; HOMA-IR = homeostatic model assessment for insulin resistance. Bolded values indicate participant characteristics that differ significantly between the stratified adiposity and/or insulin groups, where * and *** = *p*’s ≤ 0.05 and ≤0.005, respectively.

**Table 2:** Comparisons of event-related potentials by adiposity and insulin groups.

		Adiposity			Insulin		
ERP Components	Total	Lean	Obese	*t*-Value	Insulin-Sensitive	Insulin-Resistant	*t*-Value
**Negative−Neutral**							
*EPN*	1.7 (2.7)	1.7 (3.0)	1.6 (2.6)	*t* = 0.04	1.1 (2.6)	2.5 (2.6)	*t* = −1.37
*Early LPP*	0.9 (1.9)	0.6 (2.5)	1.1 (1.7)	*t* = −0.64	0.8 (2.1)	1.2 (1.7)	*t* = −0.47
*Middle LPP*	0.8 (2.2)	0.6 (2.2)	0.9 (2.2)	*t* = −0.38	0.7 (2.1)	1.1 (2.4)	*t* = −0.45
***Late LPP***	1.2 (1.8)	**2.4 (2.3)**	**0.8 (1.5)**	***t *** = 2.31 *	**1.8 (1.9)**	**0.3 (1.3)**	***t *** = 2.37 *
**Positive−Neutral**							
*EPN*	−0.3 (2.6)	−0.7 (2.1)	−0.2 (2.8)	*t* = −0.49	−0.8 (2.5)	0.4 (2.6)	*t* = −1.22
*Early LPP*	−0.1 (2.3)	0.6 (1.7)	−0.3 (2.5)	*t* = 0.96	0.0 (2.3)	−0.2 (2.5)	*t* = 0.26
*Middle LPP*	0.1 (2.7)	0.3 (2.0)	0.1 (2.9)	*t* = 0.17	0.2 (2.5)	0.0 (3.1)	*t* = 0.13
*Late LPP*	0.7 (2.2)	1.9 (2.1)	0.3 (2.2)	*t* = 1.87	1.2 (1.9)	−0.1 (2.6)	*t* = 1.69
**Negative−Positive**							
*EPN*	2.0 (2.7)	2.4 (3.1)	1.8 (2.6)	*t* = 0.50	1.9 (2.9)	2.1 (2.5)	*t* = −0.18
***Early LPP***	1.0 (1.6)	**0.0 (1.6)**	**1.4 (1.4)**	***t *** = −2.38 *	0.8 (1.7)	1.3 (1.4)	*t* = −0.97
*Middle LPP*	0.7 (2.0)	0.3 (1.2)	0.9 (2.2)	*t* = −0.65	0.5 (1.9)	1.0 (2.2)	*t* = −0.68
*Late LPP*	0.5 (2.0)	0.5 (2.0)	0.5 (2.0)	*t* = 0.03	0.6 (2.0)	0.4 (2.0)	*t* = 0.19

Values are Mean (Standard Deviation) unless otherwise indicated. In each contrasted valence condition, the mean ERP amplitudes of a Neutrally or oppositely valenced condition were subtracted from the mean ERP amplitudes of an emotionally valenced condition (i.e., Negative or Positive) as follows: (1) Negative−Neutral; (2) Positive−Neutral; and (3) Negative−Positive. For the EPN component, lower (i.e., more negative) difference score values reflect overall larger amplitude deflections in response to the emotionally valenced condition compared to the Neutrally or oppositely valenced condition. For the LPP component, higher (i.e., more positive) difference scores indicate overall larger amplitude deflections in response to the emotionally valenced condition compared to the Neutrally or oppositely valenced condition. For the stratified adiposity groups, *t*-values reflect differences between participants with and without obesity based on a body fat percentage of ≥25% (males) and ≥35% (females) versus <25% (males) and <35% (females), respectively. For the stratified insulin groups, *t*-values reflect differences between participants with insulin resistance and insulin sensitivity based on HOMA-IR values of ≥2.0 versus <2.0, respectively. Amplitude was measured in microvolts (µV). EPN = early posterior negativity; ERP = event-related potential; LPP = late positive potential. Bolded values indicate ERP amplitudes that differ significantly between the stratified adiposity and/or insulin groups, where * = *p* ≤ 0.05.

**Table 3:** Comparisons of affective processing task scores by adiposity and insulin groups.

		Adiposity			Insulin		
Affective Processing Parameters	Total	Lean	Obese	*t* Value	Insulin Sensitive	Insulin Resistant	*t*-Value
Rating (Negative−Neutral)	1.6 (0.4)	1.4 (0.4)	1.6 (0.3)	*t* = −1.23	1.6 (0.4)	1.6 (0.3)	*t* = −0.02
**RT (Negative−Neutral)**	12.6 (202.7)	32.5 (124.4)	5.4 (226.7)	*t* = 0.32	**−53.4 (184.5)**	**111.7 (194.6)**	***t *** = −2.35 *
Rating (Positive−Neutral)	−0.5 (0.2)	−0.6 (0.2)	−0.5 (0.2)	*t* = −0.67	−0.5 (0.2)	−0.5 (0.2)	*t* = −0.54
RT (Positive−Neutral)	39.4 (178.4)	65.0 (214.9)	30.2 (168.0)	*t* = 0.47	23.2 (216.7)	63.8 (101.5)	*t* = −0.69
Rating (Negative−Positive)	1.2 (0.4)	1.0 (0.6)	1.3 (0.3)	*t* = −1.38	1.2 (0.5)	1.3 (0.3)	*t* = −0.81
RT (Negative−Positive)	−26.8 (223.9)	−32.5 (162.5)	−24.8 (245.8)	*t* = −0.08	−76.6 (200.3)	47.9 (245.0)	*t* = −1.53

Values are Mean (Standard Deviation) unless otherwise indicated. In each contrasted valence condition, the affective processing parameters of a Neutrally or oppositely valenced condition were subtracted from the affective processing parameters of an emotionally valenced condition (i.e., Negative or Positive) as follows: (1) Negative−Neutral; (2) Positive−Neutral; and (3) Negative−Positive. For the contrasted oppositely valenced picture condition, valence ratings were calculated as: Negative − (4 − Positive). For the contrasted mean valence rating parameters, larger positive difference scores (i.e., higher numerical values) in the contrasted Negative−Neutral and Negative−Positive conditions indicate a greater magnitude of unpleasantness given via subjective ratings to the visual stimuli overall, whereas less negative difference scores (i.e., higher numerical values) in the contrasted Positive−Neutral condition reflect a greater magnitude of pleasantness given via subjective ratings to the visual stimuli overall. For the contrasted mean stimulus-to-response-onset RT parameters, difference scores with negative values represent faster RTs to the emotional condition versus the Neutrally or oppositely valenced condition, whereas difference scores with positive values instead reflect faster RTs to the Neutrally or oppositely valenced condition. For the stratified adiposity groups, *t*-values reflect differences between participants with and without obesity based on a body fat percentage of ≥25% (males) and ≥35% (females) versus <25% (males) and <35% (females), respectively. For the stratified insulin groups, *t*-values reflect differences between participants with insulin resistance and insulin sensitivity based on HOMA-IR values of ≥2.0 versus <2.0, respectively. Reaction time was measured in milliseconds. RT = reaction time. Bolded values indicate affective processing parameters (i.e., valence ratings, stimulus-to-response-onset reaction times) that differ significantly between the stratified adiposity and/or insulin groups, where * = *p* ≤ 0.05.

**Table 4:** Correlation matrix of event-related potentials and affective processing parameters.

	Negative−Neutral		Positive−Neutral		Negative−Positive	
ERP Components	Valence Rating	Reaction Time	Valence Rating	Reaction Time	Valence Rating	Reaction Time
**Negative−Neutral**						
*EPN*	−0.19	0.14	-	-	-	-
*Early LPP*	0.06	−0.04	-	-	-	-
*Middle LPP*	−0.02	0.04	-	-	-	-
*Late LPP*	−0.33	0.03	-	-	-	-
**Positive−Neutral**						
*EPN*	-	-	−0.21	0.12	-	-
*Early LPP*	-	-	0.00	0.10	-	-
*Middle LPP*	-	-	−0.01	0.15	-	-
*Late LPP*	-	-	−0.20	−0.05	-	-
**Negative−Positive**						
*EPN*	-	-	-	-	0.13	−0.18
*Early LPP*	-	-	-	-	0.10	−0.14
*Middle LPP*	-	-	-	-	0.13	−0.31
*Late LPP*	-	-	-	-	−0.26	−0.26

Values show bivariate Pearson correlation coefficients between the independent (EPN and LPP amplitude deflections) and dependent (valence ratings, stimulus-to-response-onset reaction times) variables for each contrasted condition during the International Affective Picture System task. In each contrasted valence condition, the mean ERP voltage amplitudes and affective processing parameters of a Neutrally or oppositely valenced condition were subtracted from the mean ERP amplitudes and affective processing parameters of an emotionally valenced condition (i.e., Negative or Positive), respectively, as follows: (1) Negative−Neutral; (2) Positive−Neutral; and (3) Negative−Positive. For the contrasted oppositely valenced picture condition, valence ratings were calculated as: Negative − (4 − Positive). Amplitude was measured in microvolts (µV); reaction time was measured in milliseconds. EPN = early posterior negativity; ERP = event-related potential; LPP = late positive potential.

**Table 5:** Hierarchical moderated regression results for Negative vs. Neutral valence ratings.

	Step 1					Step 2				
Variables	*β*	*t*	*R* ^2^	*F*	*p*	*β*	*t*	*R*^2^/Δ*R*^2^	*F*/Δ*F*	*p*
**Model 1**			0.12	1.77	0.19			0.13	1.26	0.31
*EPN*	−0.18	−1.01				−0.20	−1.06			
*BF%*	0.28	1.55				0.28	1.51			
*EPN × BF%*						0.11	0.57	0.01	0.33	0.57
**Model 2**			0.05	0.64	0.53			0.05	0.48	0.70
*EPN*	−0.19	−0.98				−0.18	−0.91			
*HOMA-IR*	0.09	0.48				0.09	0.46			
*EPN × HOMA-IR*						0.09	0.45	0.01	0.20	0.66
**Model 3**			0.09	1.35	0.28			0.10	0.92	0.44
*eLPP*	0.09	0.51				0.08	0.40			
*BF%*	0.30	1.62				0.28	1.49			
*eLPP × BF%*						−0.08	−0.39	0.01	0.15	0.70
**Model 4**			0.01	0.19	0.83			0.01	0.12	0.95
*eLPP*	0.05	0.24				0.04	0.23			
*HOMA-IR*	0.10	0.53				0.10	0.53			
*eLPP × HOMA-IR*						−0.02	−0.11	0.00	0.01	0.91
**Model 5**			0.08	1.23	0.31			0.09	0.84	0.48
*mLPP*	−0.04	−0.21				−0.03	−0.17			
*BF%*	0.29	1.57				0.30	1.58			
*mLPP × BF%*						0.07	0.37	0.01	0.13	0.72
**Model 6**			0.01	0.17	0.85			0.03	0.27	0.85
*mLPP*	−0.03	−0.15				−0.04	−0.22			
*HOMA-IR*	0.11	0.57				0.08	0.37			
*mLPP × HOMA-IR*						0.14	0.69	0.02	0.47	0.50
**Model 7**			0.14	2.11	0.14			0.40 ***	5.85 ***	**0.003**
*lLPP*	−0.25	−1.28				0.10	0.50			
*BF%*	0.18	0.91				0.16	0.94			
*lLPP × BF% *						0.63 ***	3.41 ***	0.27 ***	11.66 ***	**0.002**
**Model 8**			0.11	1.64	0.23			0.34	4.42	0.012
*lLPP*	−0.33	−1.71				−0.11	−0.60			
*HOMA-IR*	0.00	0.00				0.39	0.07			
*lLPP × HOMA-IR*						0.52 **	3.00 **	0.23 **	9.01 **	**0.006**

Table displays the results of each step of the hierarchical moderated regression analyses, which tested whether the relationships between EPN or LPP voltage amplitudes and mean contrasted valence ratings after viewing Negatively valenced pictures versus Neutrally valenced pictures were moderated by BF% or HOMA-IR values. A significant interaction term in step 2 (i.e., significant Δ*R*^2^ and Δ*F* terms) determines the presence of a moderation effect. *β* shows standardized regression coefficients. Only statistically significant covariates (*p* ≤ 0.10) were included in each model. BF% = body fat percentage; eLPP = late positive potential in the early latency window; EPN = early posterior negativity; HOMA-IR = homeostatic model assessment for insulin resistance; lLPP = late positive potential in the late latency window; mLPP = late positive potential in the middle latency window. Bolded values indicate statistical significance, where ** and *** = *p*’s ≤ 0.0063 (Bonferroni corrected) and ≤0.005, respectively.

**Table 6:** Hierarchical moderated regression results for Negative vs. Neutral reaction times.

	Step 1					Step 2				
Variables	*β*	*t*	*R* ^2^	*F*	*p*	*β*	*t*	*R*^2^/Δ*R*^2^	*F*/Δ*F*	*p*
**Model 1**			0.16	1.67	0.20			0.20	1.60	0.21
*EPN*	0.12	0.68				0.15	0.82			
*BF%*	0.01	0.05				0.01	0.07			
*Age*	0.38	2.07				0.39	2.18			
*EPN × BF%*						−0.21	−1.16	0.04	1.35	0.26
**Model 2**			0.24	3.27	0.03			0.28	2.40	0.08
*EPN*	0.03	0.16				0.13	0.74			
*HOMA-IR*	0.40	2.29				0.30	1.69			
*Age*	0.32	1.93				0.36	2.00			
*Race/Ethnicity*	0.35	1.93								
*EPN × HOMA-IR*						−0.18	−1.04	0.03	1.09	0.31
**Model 3**			0.15	1.54	0.23			0.15	1.11	0.37
*eLPP*	0.07	0.36				0.06	0.32			
*BF%*	0.01	0.06				0.01	0.03			
*Age*	0.40	2.11				0.40	2.07			
*eLPP × BF%*						−0.02	−0.11	0.00	0.01	0.91
**Model 4**			0.34	3.30	0.03			0.40	3.16	0.03
*eLPP*	−0.02	−0.12				−0.03	−0.21			
*HOMA-IR*	0.40	2.27				0.39	2.27			
*Age*	0.32	1.83				0.34	2.00			
*Race/Ethnicity*	0.37	2.13				0.33	1.96			
*eLPP × HOMA-IR*						−0.24	−1.46	0.05	2.13	0.16
**Model 5**			0.16	1.66	0.20			0.17	1.23	0.32
*mLPP*	0.12	0.68				0.12	0.64			
*BF%*	−0.01	−0.05				−0.02	−0.11			
*Age*	0.41	2.20				0.41	2.17			
*mLPP × BF%*						−0.06	−0.32	0.00	0.10	0.75
**Model 6**			0.35	3.34	0.03			0.36	2.70	0.05
*mLPP*	0.07	0.45				0.09	0.53			
*HOMA-IR*	0.39	2.20				0.41	2.25			
*Age*	0.34	2.00				0.37	2.08			
*Race/Ethnicity*	0.36	2.13				0.35	2.01			
*mLPP × HOMA-IR*						−0.12	−0.67	0.01	0.45	0.51
**Model 7**			0.26	2.22	0.10			0.36	2.68	0.05
*lLPP*	0.28	1.32				0.09	0.40			
*BF%*	0.23	1.09				0.27	1.30			
*Age*	0.40	2.29				0.41	2.48			
*Race/Ethnicity*	0.39	1.93				0.43	2.22			
*lLPP × BF%*						−0.38	−1.89	0.10	3.58	0.07
**Model 8**			0.47 ***	5.43 ***	**0.003**			0.53 ***	5.35 ***	**0.002**
*lLPP*	0.41	2.39				0.28	1.57			
*HOMA-IR*	0.58 ***	3.33 ***				0.54 ***	3.17 ***			
*Age*	0.33	2.23				0.32	2.24			
*Race/Ethnicity*	0.54 ***	3.16 ***				0.51 ***	3.11 ***			
*lLPP × HOMA-IR*						−0.27	−1.77	0.06	3.15	0.09

Table displays the results of each step of the hierarchical moderated regression analyses, which tested whether the relationships between EPN or LPP voltage amplitudes and mean contrasted stimulus-to-response-onset reaction times after viewing Negatively valenced pictures versus Neutrally valenced pictures were moderated by BF% or HOMA-IR values. A significant interaction term in step 2 (i.e., significant Δ*R*^2^ and Δ*F* terms) determines the presence of a moderation effect. *β* shows standardized regression coefficients. Only statistically significant covariates (*p* ≤ 0.10) were included in each model. BF% = body fat percentage; eLPP = early latency late positive potential; EPN = early posterior negativity; HOMA-IR = homeostatic model assessment for insulin resistance; lLPP = late latency late positive potential; mLPP = middle latency late positive potential. Bolded values indicate statistical significance, where *** = *p* ≤ 0.005.

**Table 7:** Hierarchical moderated regression results for Positive vs. Neutral valence ratings.

	Step 1					Step 2				
Variables	*β*	*t*	*R* ^2^	*F*	*p*	*β*	*t*	*R*^2^/Δ*R*^2^	*F*/Δ*F*	*p*
**Model 1**			0.59 ***	6.85 ***	**<0.001**			0.59 ***	5.48 ***	**0.001**
*EPN*	−0.16	−1.21				−0.16	−1.11			
*BF%*	0.45	2.67				0.45	2.61			
*Age*	−0.26	−1.91				−0.26	−1.87			
*Physical Activity*	−0.29	−1.79				−0.29	−1.76			
*Race/Ethnicity*	0.73 ***	5.12 ***				0.73 ***	5.00 ***			
*EPN × BF%*						−0.02	−0.13	0.00	0.02	0.90
**Model 2**			0.51 ***	6.55 ***	**<0.001**			0.52 ***	5.09 ***	0.003 ***
*EPN*	−0.22	−1.56				−0.22	−1.55			
*HOMA-IR*	0.24	1.57				0.24	1.53			
*Age*	−0.30	−2.07				−0.29	−2.01			
*Race/Ethnicity*	0.65 ***	4.44 ***				0.65 ***	4.30 ***			
*EPN × HOMA-IR*						0.06	0.40	0.00	0.16	0.69
**Model 3**			0.57 ***	6.41 ***	**<0.001**			**0.52**	5.18 ***	**0.002**
*eLPP*	−0.11	−0.71				−0.15	−0.95			
*BF%*	0.43	2.38				0.24	1.54			
*Age*	−0.30	−2.10				−0.34	−2.31			
*Physical Activity*	−0.29	−1.76								
*Race/Ethnicity*	0.73 ***	5.07 ***				0.67 ***	4.48 ***			
*eLPP × BF%*						0.04	0.28	0.00	0.08	0.79
**Model 4**			0.50 ***	6.24 ***	**0.001**			0.51 ***	4.99 ***	**0.003**
*eLPP*	−0.20	−1.34				−0.23	−1.45			
*HOMA-IR*	0.20	1.29				0.21	1.36			
*Age*	−0.36	−2.41				−0.36	−2.41			
*Race/Ethnicity*	0.67 ***	4.45 ***				0.68 ***	4.45 ***			
*eLPP × HOMA-IR*						0.11	0.71	0.01	0.50	0.49
**Model 5**			0.52 ***	6.62 ***	**<0.001**			0.58 ***	5.37 ***	**0.001**
*mLPP*	−0.12	−0.87				0.00	0.02			
*BF%*	0.27	1.84				0.45	2.51			
*Age*	−0.33	−2.30				−0.27	−1.94			
*Physical Activity*						−0.34	−1.89			
*Race/Ethnicity*	0.67 ***	4.58 ***				0.72 ***	4.94 ***			
*mLPP × BF%*						−0.15	−0.98	0.02	0.97	0.34
**Model 6**			0.49 ***	5.95 ***	**0.002**			0.49 ***	4.67 ***	**0.004**
*mLPP*	−0.16	−1.07				−0.13	−0.83			
*HOMA-IR*	0.21	1.38				0.22	1.40			
*Age*	−0.34	−2.28				−0.34	−2.25			
*Race/Ethnicity*	0.66 ***	4.35 ***				0.65 ***	4.17 ***			
*mLPP × HOMA-IR*						−0.08	−0.53	0.01	0.28	0.60
**Model 7**			0.59 ***	6.98 ***	**<0.001**			0.60 ***	5.79 ***	**<0.001**
*lLPP*	−0.20	−1.32				−0.23	−1.45			
*BF%*	0.39	2.23				0.36	1.93			
*Age*	−0.32	−2.31				−0.31	−2.20			
*Physical Activity*	−0.33	−2.06				−0.35	−2.12			
*Race/Ethnicity*	0.69 ***	4.78 ***				0.66 ***	4.45 ***			
*lLPP × BF%*						0.11	0.74	0.01	0.54	0.47
**Model 8**			0.51 ***	6.39 ***	**0.001**			0.51 ***	4.91 ***	**0.003**
*lLPP*	−0.22	−1.45				−0.22	−1.35			
*HOMA-IR*	0.15	0.95				0.15	0.93			
*Age*	−0.37	−2.48				−0.37	−2.43			
*Race/Ethnicity*	0.60 ***	3.99 ***				0.60 ***	3.91 ***			
*lLPP × HOMA-IR*						−0.01	−0.05	0.00	0.00	0.96

Table displays the results of each step of the hierarchical moderated regression analyses, which tested whether the relationships between EPN or LPP voltage amplitudes and mean contrasted valence ratings after viewing Positively valenced pictures versus Neutrally valenced pictures were moderated by BF% or HOMA-IR values. A significant interaction term in step 2 (i.e., significant Δ*R*^2^ and Δ*F* terms) determines the presence of a moderation effect. *β* shows standardized regression coefficients. Only statistically significant covariates (*p* ≤ 0.10) were included in each model. BF% = body fat percentage; eLPP = early latency late positive potential; EPN = early posterior negativity; HOMA-IR = homeostatic model assessment for insulin resistance; lLPP = late latency late positive potential; mLPP = middle latency late positive potential. Bolded values indicate statistical significance, where *** = *p* ≤ 0.005.

**Table 8:** Hierarchical moderated regression results for Positive vs. Neutral reaction times.

	Step 1					Step 2				
Variables	*β*	*t*	*R* ^2^	*F*	*p*	*β*	*t*	*R*^2^/Δ*R*^2^	*F/*Δ*F*	*p*
**Model 1**			0.03	0.34	0.71			0.08	0.74	0.54
*EPN*	0.12	0.63				0.17	0.90			
*BF%*	−0.10	−0.52				−0.09	−0.48			
*EPN × BF%*						−0.24	−1.24	0.05	1.53	0.23
**Model 2**			0.02	0.22	0.80			0.04	0.33	0.80
*EPN*	0.13	0.66				0.13	0.69			
*HOMA-IR*	−0.03	−0.16				−0.03	−0.17			
*EPN × HOMA-IR*						−0.14	−0.74	0.02	0.55	0.47
**Model 3**			0.02	0.21	0.81			0.02	0.14	0.94
*eLPP*	0.08	0.38				0.08	0.38			
*BF%*	−0.08	−0.37				−0.07	−0.36			
*eLPP × BF%*						−0.02	−0.09	0.00	0.01	0.93
**Model 4**			0.01	0.14	0.87			0.04	0.39	0.77
*eLPP*	0.10	0.53				0.15	0.73			
*HOMA-IR*	0.00	0.01				−0.02	−0.11			
*eLPP × HOMA-IR*						−0.19	−0.93	0.03	0.87	0.36
**Model 5**			0.03	0.39	0.68			0.04	0.33	0.81
*mLPP*	0.13	0.70				0.17	0.80			
*BF%*	−0.08	−0.42				−0.10	−0.51			
*mLPP × BF%*						−0.10	−0.48	0.01	0.23	0.63
**Model 6**			0.02	0.30	0.75			0.08	0.76	0.52
*mLPP*	0.15	0.77				0.23	1.15			
*HOMA-IR*	−0.01	−0.04				0.03	0.13			
*mLPP × HOMA-IR*						−0.26	−1.30	0.06	1.68	0.21
**Model 7**			0.02	0.29	0.75			0.02	0.22	0.89
*lLPP*	−0.11	−0.54				−0.13	−0.58			
*BF%*	−0.15	−0.71				−0.17	−0.75			
*lLPP × BF% *						0.06	0.30	0.00	0.09	0.77
**Model 8**			0.00	0.05	0.95			0.02	0.19	0.90
*lLPP*	−0.06	−0.30				−0.02	−0.08			
*HOMA-IR*	−0.03	−0.15				−0.02	−0.08			
*lLPP × HOMA-IR*						−0.14	−0.69	0.02	0.47	0.50

Table displays the results of each step of the hierarchical moderated regression analyses, which tested whether the relationships between EPN or LPP voltage amplitudes and mean contrasted stimulus-to-response-onset reaction times after viewing Positively valenced pictures versus Neutrally valenced pictures were moderated by BF% or HOMA-IR values. A significant interaction term in step 2 (i.e., significant Δ*R*^2^ and Δ*F* terms) determines the presence of a moderation effect. *β* shows standardized regression coefficients. Only statistically significant covariates (*p* ≤ 0.10) were included in each model. BF% = body fat percentage; eLPP = early latency late positive potential; EPN = early posterior negativity; HOMA-IR = homeostatic model assessment for insulin resistance; lLPP = late latency late positive potential; mLPP = middle latency late positive potential.

**Table 9:** Hierarchical moderated regression results for Negative vs. Positive valence ratings.

	Step 1					Step 2				
Variables	*β*	*t*	*R* ^2^	*F*	*p*	*β*	*t*	*R*^2^/Δ*R*^2^	*F*/Δ*F*	*p*
**Model 1**			0.23	2.53	0.08			0.24	2.02	0.12
*EPN*	0.08	0.45				0.07	0.39			
*BF%*	0.53	2.58				0.53	2.56			
*Physical Activity*	−0.40	−1.92				−0.40	−1.92			
*EPN × BF%*						0.13	0.77	0.02	0.60	0.45
**Model 2**			0.06	0.82	0.45			0.09	0.84	0.49
*EPN*	0.17	0.90				0.22	1.11			
*HOMA-IR*	0.21	1.08				0.21	1.09			
*EPN × HOMA-IR*						0.18	0.94	0.03	0.88	0.36
**Model 3**			0.22	2.45	0.09			0.24	2.02	0.12
*eLPP*	−0.03	−0.14				−0.05	−0.28			
*BF%*	0.55	2.52				0.53	2.43			
*Physical Activity*	−0.41	−2.01				−0.42	−2.04			
*eLPP × BF%*						−0.16	−0.89	0.02	0.80	0.38
**Model 4**			0.03	0.44	0.65			0.27	1.79	0.15
*eLPP*	0.05	0.25				−0.10	−0.53			
*HOMA-IR*	0.15	0.76				0.38	1.77			
*Physical Activity*						−0.35	−1.75			
*Sex*						−0.40	−2.16			
*eLPP × HOMA-IR*						−0.30	−1.56	0.07	2.43	0.13
**Model 5**			0.22	2.45	0.09			0.22	1.78	0.16
*mLPP*	0.02	0.14				0.02	0.12			
*BF%*	0.53	2.48				0.52	2.36			
*Physical Activity*	−0.41	−2.00				−0.41	−1.93			
*mLPP × BF%*						0.04	0.22	0.00	0.05	0.83
**Model 6**			0.04	0.53	0.59			0.05	0.44	0.73
*mLPP*	0.10	0.50				0.12	0.61			
*HOMA-IR*	0.15	0.79				0.17	0.86			
*mLPP × HOMA-IR*						−0.10	−0.52	0.01	0.27	0.61
**Model 7**			0.30	3.79	0.02			0.38	3.81	0.02
*lLPP*	−0.29	−1.77				−0.28	−1.79			
*BF%*	0.56	2.88				0.55	2.96			
*Physical Activity*	−0.42	−2.15				−0.41	−2.17			
*lLPP × BF%*						0.27	1.73	0.08	3.00	0.10
**Model 8**			0.25	2.13	0.11			0.24	1.93	0.14
*lLPP*	−0.24	−1.37				−0.34	−1.89			
*HOMA-IR*	0.28	1.43				0.05	0.25			
*Physical Activity*	−0.34	−1.71								
*Sex*	−0.34	−1.91								
*Race/Ethnicity*						−0.39	−2.09			
*lLPP × HOMA-IR*						0.12	0.68	0.01	0.46	0.50

Table displays the results of each step of the hierarchical moderated regression analyses, which tested whether the relationships between EPN or LPP voltage amplitudes and mean contrasted valence ratings after viewing Negatively valenced pictures versus Positively valenced pictures were moderated by BF% or HOMA-IR values. A significant interaction term in step 2 (i.e., significant ΔR*^2^* and Δ*F* terms) determines the presence of a moderation effect. *β* shows standardized regression coefficients. Only statistically significant covariates (*p* ≤ 0.10) were included in each model. BF% = body fat percentage; eLPP = early latency late positive potential; EPN = early posterior negativity; HOMA-IR = homeostatic model assessment for insulin resistance; lLPP = late latency late positive potential; mLPP = middle latency late positive potential.

**Table 10:** Hierarchical moderated regression results for Negative vs. Positive reaction times.

	Step 1					Step 2				
Variables	*β*	*t*	*R* ^2^	*F*	*p*	*β*	*t*	*R*^2^/Δ*R*^2^	*F*/Δ*F*	*p*
**Model 1**			0.24	2.79	0.06			0.25	2.03	0.12
*EPN*	−0.31	−1.81				−0.31	−1.75			
*BF%*	0.08	0.49				0.08	0.47			
*Age*	0.35	2.00				0.35	1.97			
*EPN × BF%*						−0.04	−0.20	0.00	0.04	0.84
**Model 2**			0.35	3.41	0.02			0.29	2.55	0.06
*EPN*	−0.36	−2.06				−0.33	−1.79			
*HOMA-IR*	0.29	1.64				0.37	2.05			
*Age*	0.31	1.91								
*Race/Ethnicity*	0.31	1.72				0.36	1.88			
*EPN × HOMA-IR*						0.19	1.05	0.03	1.11	0.30
**Model 3**			0.19	2.00	0.14			0.24	1.97	0.13
*eLPP*	−0.21	−1.11				−0.17	−0.91			
*BF%*	0.16	0.82				0.19	1.02			
*Age*	0.36	2.03				0.31	1.71			
*eLPP × BF%*						0.24	1.31	0.05	1.73	0.20
**Model 4**			0.28	3.29	0.04			0.28	3.30	0.04
*eLPP*	−0.27	−1.55				−0.18	−0.97			
*HOMA-IR*	0.36	1.97				0.31	1.66			
*Age*	0.31	1.84								
*eLPP × HOMA-IR*						0.33	1.85	0.10	3.42	0.08
**Model 5**			0.15	1.52	0.23			0.16	1.22	0.33
*mLPP*	0.00	0.02				−0.01	−0.04			
*BF%*	0.08	0.42				0.07	0.34			
*Age*	0.37	1.99				0.35	1.84			
*mLPP × BF%*						0.12	0.65	0.01	0.42	0.52
**Model 6**			0.21	2.29	0.10			0.24	2.74	0.06
*mLPP*	−0.02	−0.13				−0.14	−0.76			
*HOMA-IR*	0.27	1.47				0.28	1.58			
*Age*	0.32	1.80								
*mLPP × HOMA-IR*						0.38	2.11	0.13	4.44	0.05
**Model 7**			0.17	1.72	0.19			0.17	1.28	0.30
*lLPP*	−0.13	−0.73				−0.13	−0.68			
*BF%*	0.09	0.48				0.09	0.49			
*Age*	0.40	2.13				0.38	1.94			
*lLPP × BF%*						0.07	0.36	0.00	0.13	0.72
**Model 8**			0.22	2.46	0.09			0.23	1.90	0.14
*lLPP*	−0.12	−0.65				−0.12	−0.64			
*HOMA-IR*	0.26	1.44				0.26	1.46			
*Age*	0.35	1.93				0.33	1.78			
*lLPP × HOMA-IR*						0.11	0.64	0.01	0.40	0.53

Table displays the results of each step of the hierarchical moderated regression analyses, which tested whether the relationships between EPN or LPP voltage amplitudes and mean contrasted stimulus-to-response-onset reaction times after viewing Negatively valenced pictures versus Positively valenced pictures were moderated by BF% or HOMA-IR values. A significant interaction term in step 2 (i.e., significant Δ*R*^2^ and Δ*F* terms) determines the presence of a moderation effect. *β* shows standardized regression coefficients. Only statistically significant covariates (*p* ≤ 0.10) were included in each model. BF% = body fat percentage; eLPP = early latency late positive potential; EPN = early posterior negativity; HOMA-IR = homeostatic model assessment for insulin resistance; lLPP = late latency late positive potential; mLPP = middle latency late positive potential.

## Data Availability

The data presented in this study are available on request from the corresponding author due to privacy and ethical reasons.

## Supplementary Materials

The following supporting information can be downloaded at: https://www.mdpi.com/article/10.3390/brainsci16010003/s1, Table S1: Key demographic comparisons between included versus excluded participants; Table S2: Comparisons of event-related potentials for each separate valence condition by adiposity and insulin groups; Table S3: Comparisons of affective processing task scores for each separate valence condition by adiposity and insulin groups; Table S4: Correlation matrix of event-related potentials and affective processing parameters for each separate valence condition; Figure S1: International Affective Picture System (IAPS) task procedure; Figures S2a–f: Non-differenced Negative, Neutral, and Positive EPN and LPP waveforms by body fat percentage; Figures S3a–f: Non-differenced Negative, Neutral, and Positive EPN and LPP waveforms by insulin resistance levels.

## Abbreviations

The following abbreviations are used in this manuscript:

ACC	Anterior cingulate cortex
AG	Angular gyrus
aINS	Anterior insula
BF%	Body fat percentage
BG	Basal ganglia
BMI	Body mass index
BP	Blood pressure
DXA	Dual-energy X-ray absorptiometry scan
dlPFC	Dorsolateral prefrontal cortex
EEG	Electroencephalography
eLPP	Late positive potential in the early latency window
EPN	Early posterior negativity
ERP	Event-related potential
fMRI	Functional magnetic resonance imaging
Hemoglobin A1c	Glycated hemoglobin
HOMA-IR	Homeostatic model assessment for insulin resistance
IR	Insulin resistance/resistant
IS	Insulin sensitivity/sensitive
lLPP	Late positive potential in the late latency window
LPP	Late positive potential
mLPP	Late positive potential in the middle latency window
ms	Milliseconds
OFC	Orbitofrontal cortex
PFC	Prefrontal cortex
RT	Reaction time
SMA	Supplementary motor area
STG	Superior temporal gyrus
vlPFC	Ventrolateral prefrontal cortex

## Appendix A

**Table A1:** International Affective Picture System task photo identification numbers by valence condition.

Negative	Neutral	Positive
3030, 3060, 3071, 3100, 3102, 3130, 3150, 3170, 6020, 6230, 6313, 6570, 9040, 9140, 9181, 9253, 9265, 9320, 9400, 9420, 9433, 9570, 9571	2372, 2383, 2514, 2840, 5410, 6150, 6900, 7000, 7002, 7006, 7009, 7010, 7035, 7080, 7130, 7170, 7175, 7233, 7491, 7550, 7705	1340, 1463, 2165, 2306, 2374, 4603, 4611, 4652, 4656, 4810, 5202, 5611, 5764, 6250.2, 7200, 7250, 7508, 8118, 8251, 8500

Table displays the photo identification number for each photo used in the Negatively, Neutrally, and Positively valenced picture conditions during the International Affective Picture System task.
