# Supplementary material for: Obesity and Insulin Resistance Alter Neural Processing of Unpleasant, but Not Pleasant, Visual Stimuli in Young Adults
Source: Brain Sci. 2025 Dec 19;16(1):3. doi: 10.3390/brainsci16010003 (PMC12839355; doi:10.3390/brainsci16010003)
Supplement: Supplementary file 1 [file brainsci-16-00003-s001.zip › brainsci-4032316_Supplementary.pdf]

Supplementary Table S1. Participant characteristic comparisons between included and excluded participants

Data (unit)	Included Participants (*N*=30)	Excluded Participants (*N*=30)	*t*-value/chi-square
Age (years)	25.7 (5.3)	24.3 (4.9)	*t* = −1.02
Sex (*n* females (%))	15 (50.0%)	18 (60.0%)	$\chi 2$ = 0.61
Activity Level (*n* (%))			$\chi 2$ = 3.30
Sedentary/Low Active	20 (66.7%)	13 (43.3%)	
Active/Very Active	10 (33.3%)	17 (56.7%)	
Race/Ethnicity (*n* (%))			$\chi 2$ = 5.95
White	24 (80.0%)	17 (56.7%)	
Asian	5 (16.7%)	6 (20.0%)	
Hispanic/Latinx	1 (3.3%)	3 (10.0%)	
Black	0 (0.0%)	4 (13.3%)	
BF% (DXA)	37.1 (10.3)	33.4 (9.2)	*t* = −1.46
BMI	29.3 (8.2)	26.2 (7.0)	*t* = −1.53
BP, diastolic (kPa)	10.2 (1.7)	9.9 (1.4)	*t* = −0.71
BP, systolic (kPa)	16.2 (2.1)	15.4 (2.0)	*t* = −1.52
Glucose (mmol/L), fasting	5.0 (0.5)	5.3 (2.1)	*t* = 0.79
Height (meters)	1.7 (0.1)	1.7 (0.1)	*t* = −0.87
Hemoglobin A1c (%)	5.4 (0.2)	5.4 (1.3)	*t* = 0.36
HOMA-IR	2.6 (2.5)	2.6 (6.6)	*t* = −0.05
Insulin (pmol/L), fasting	69.3 (63.0)	58.5 (130.7)	*t* = −0.41

Values indicate Mean (Standard Deviation) unless otherwise noted. Table shows comparisons of key characteristics between study participants who were included in the final analyses and participants who were excluded from the final analyses due to attrition (*n*=24) and missing (*n*=5) or excessively noisy (*n*=1) EEG signals, which were tested utilizing independent-sample *t*-tests, chi-square, or Fisher's exact tests. BF% = body fat percentage; BMI = body mass index; BP = blood pressure; DXA = dual-energy X-ray absorptiometry; hemoglobin A1c = glycated hemoglobin; HOMA-IR = homeostatic model assessment for insulin resistance.

Supplementary Table S2. Comparisons of event-related potentials for each separate valence condition by adiposity and insulin groups

ERP Components	Total	Adiposity			Insulin		
		Lean	Obese	*t*-value	Insulin-sensitive	Insulin-resistant	*t*-value
**Negative**							
EPN	4.3 (2.8)	4.7 (4.2)	4.2 (2.2)	*t* = 0.55	3.7 (3.0)	5.2 (2.3)	*t* = −1.44
Early LPP	4.2 (2.6)	4.3 (2.9)	4.1 (2.6)	*t* = 0.14	3.8 (2.4)	4.7 (2.9)	*t* = −0.97
Middle LPP	2.7 (2.8)	3.2 (2.4)	2.5 (3.0)	*t* = 0.56	2.7 (1.9)	2.7 (4.0)	*t* = −0.01
Late LPP	0.7 (2.7)	1.2 (1.2)	0.5 (3.1)	*t* = 0.62	**1.6 (2.7)**	**−0.6 (2.2)**	***t* = 2.25***
**Positive**							
EPN	2.3 (3.4)	2.4 (5.2)	2.3 (2.7)	*t* = 0.04	1.8 (3.9)	3.1 (2.6)	*t* = −1.02
Early LPP	3.2 (3.1)	4.3 (3.6)	2.7 (2.9)	*t* = 1.26	3.0 (3.1)	3.4 (3.1)	*t* = −0.32
Middle LPP	1.9 (2.9)	2.8 (3.1)	1.6 (2.9)	*t* = 0.99	2.1 (2.7)	1.7 (3.4)	*t* = 0.45
Late LPP	0.2 (2.9)	0.7 (2.6)	0.0 (3.1)	*t* = 0.59	1.0 (3.0)	−1.0 (2.5)	*t* = 1.90
**Neutral**							
EPN	2.6 (3.3)	3.0 (5.3)	2.4 (2.3)	*t* = 0.31	2.6 (4.1)	2.7 (1.7)	*t* = −0.13
Early LPP	3.2 (2.6)	3.7 (3.0)	3.0 (2.4)	*t* = 0.63	3.0 (2.4)	3.6 (2.8)	*t* = −0.62
Middle LPP	1.8 (2.8)	2.6 (2.8)	1.5 (2.8)	*t* = 0.87	2.0 (2.3)	1.6 (3.6)	*t* = 0.34
Late LPP	−0.5 (3.0)	−1.2 (2.9)	−0.3 (3.0)	*t* = −0.75	−0.3 (2.9)	−0.9 (3.1)	*t* = 0.55

Values are Mean (Standard Deviation) unless otherwise indicated. For the stratified adiposity groups, *t*-values reflect differences between participants with and without obesity based on a body fat percentage of ≥25% (males) and ≥35% (females) versus <25% (males) and <35% (females), respectively. For the stratified insulin groups, *t*-values reflect differences between participants with insulin resistance and insulin sensitivity based on HOMA-IR values of ≥2.0 versus <2.0, respectively. Amplitude was measured in microvolts (μV). EPN = early posterior negativity; ERP = event-related potential; LPP = late positive potential. Bolded values indicate ERP amplitudes that differ significantly between the stratified adiposity and/or insulin groups, where * = $p \leq 0.05$.

Supplementary Table S3. Comparisons of affective processing task scores for each separate valence condition by adiposity and insulin groups

		Adiposity			Insulin		
**Affective Processing Parameters**	**Total**	**Lean**	**Obese**	***t*-value**	**Insulin Sensitive**	**Insulin Resistant**	***t*-value**
Rating (Negative)	3.6 (0.3)	3.5 (0.5)	3.7 (0.2)	$t = -1.20$	3.6 (0.4)	3.7 (0.2)	$t = -0.35$
RT (Negative)	1060.5 (481.7)	954.2 (368.0)	1099.1 (519.0)	$t = -0.72$	1040.2 (506.8)	1090.9 (461.5)	$t = -0.28$
Rating (Positive)	1.6 (0.2)	1.5 (0.2)	1.6 (0.3)	$t = -0.86$	1.5 (0.2)	1.6 (0.3)	$t = -0.91$
RT (Positive)	1087.3 (496.1)	986.7 (315.0)	1123.9 (549.1)	$t = -0.66$	1116.8 (590.6)	1043.0 (325.9)	$t = 0.39$
Rating (Neutral)	2.1 (0.2)	2.1 (0.3)	2.1 (0.2)	$t = -0.25$	2.1 (0.3)	2.1 (0.2)	$t = -0.42$
RT (Neutral)	1047.8 (444.3)	921.7 (363.2)	1093.7 (469.4)	$t = -0.94$	1093.6 (510.2)	979.2 (331.2)	$t = 0.68$

Values are Mean (Standard Deviation) unless otherwise indicated. Valence ratings represent the mean ratings of the magnitude of pleasantness or unpleasantness of images for each separate valence condition, where 1="very positive," 2="somewhat positive," 3="somewhat negative," and 4="very negative," where higher valence ratings indicate a higher magnitude of unpleasantness assigned via subjective ratings to the presented images. Higher stimulus-to-response-onset reaction time values indicate slower reaction times. For the stratified adiposity groups, *t*-values reflect differences between participants with and without obesity based on a body fat percentage of ≥25% (males) and ≥35% (females) versus <25% (males) and <35% (females), respectively. For the stratified insulin groups, *t*-values reflect differences between participants with insulin resistance and insulin sensitivity based on HOMA-IR values of ≥2.0 versus <2.0, respectively. Reaction time was measured in milliseconds. RT = reaction time.

Supplementary Table S4. Correlation matrix of event-related potentials and affective processing parameters for each separate valence condition

ERP Components	Negative		Positive		Neutral	
	Valence Rating	Reaction Time	Valence Rating	Reaction Time	Valence Rating	Reaction Time
**Negative**						
EPN	**0.45***	−0.11	-	-	-	-
Early LPP	**0.38***	−0.29	-	-	-	-
Middle LPP	0.18	−0.17	-	-	-	-
Late LPP	−0.04	0.02	-	-	-	-
**Positive**						
EPN	-	-	−0.08	−0.29	-	-
Early LPP	-	-	−0.23	−0.24	-	-
Middle LPP	-	-	−0.25	−0.12	-	-
Late LPP	-	-	−0.09	0.00	-	-
**Neutral**						
EPN	-	-	-	-	0.05	−0.19
Early LPP	-	-	-	-	−0.10	−0.21
Middle LPP	-	-	-	-	0.08	−0.11
Late LPP	-	-	-	-	−0.07	0.21

Values show bivariate Pearson correlation coefficients between the independent (EPN and LPP amplitudes) and dependent (valence ratings, stimulus-to-response-onset reaction times) variables for each separate valence condition during the International Affective Picture System task. Amplitude was measured in microvolts (μV); reaction time was measured in milliseconds. EPN = early posterior negativity; ERP = event-related potential; LPP = late positive potential. Bolded values indicate significant direct correlations between ERP amplitudes and affective processing parameters in each separate valence condition, where * = $p \leq 0.05$.

IAPS Task

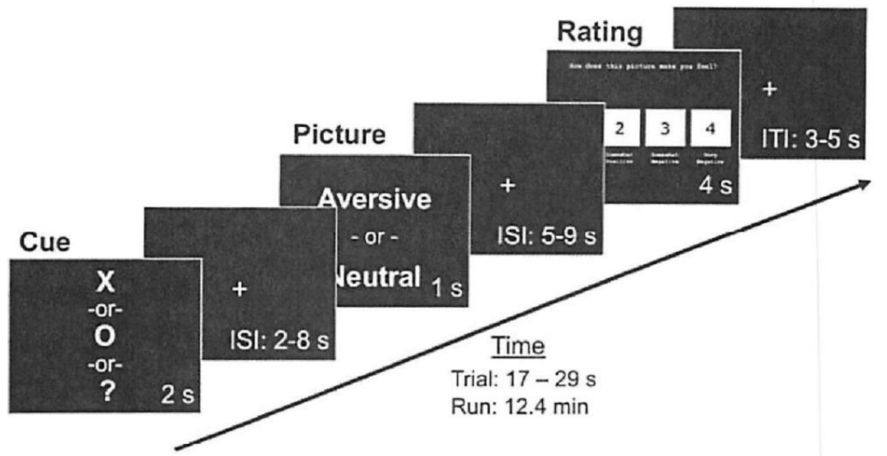

This top schematic represents trial presentation during an EEG task for the International Affective Picture System (IAPS) task. Each experimental trial (pictured) consists of a cue ("X", "O", or "?") presented for 2 seconds, followed--after a jittered inter-stimulus interval (ISI) (range: 2-8 seconds)--by a 1 s picture presentation (Aversive or Neutral). After a second jittered ISI (range: 5-9 s), subjects have 4 s to rate their emotional response to the image using a 4-item Likert scale ranging from 1 ("very positive") to 4 ("very negative"). The "X" and "O" cues indicates that the subsequent image would be aversive or neutral, respectively, whereas the "?" cue provides no information concerning the emotional content of the image.

Adapted from Motzkin et al. in Biol. Psychiatry 2015.

Supplementary Figure S1. Protocol for the International Affective Picture System (IAPS) task.

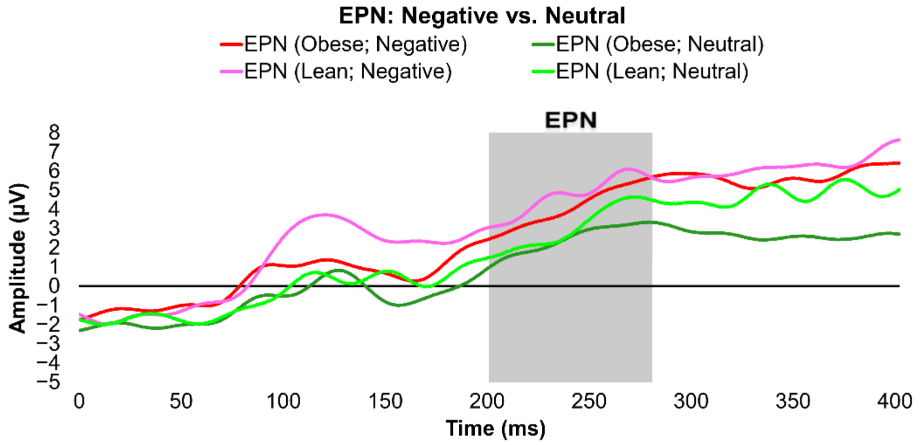
EPN: Negative vs. Neutral
EPN (Obese; Negative)
EPN (Obese; Neutral)
EPN (Lean; Negative)
EPN (Lean; Neutral)
EPN
Amplitude (μV)
Time (ms)

(a)

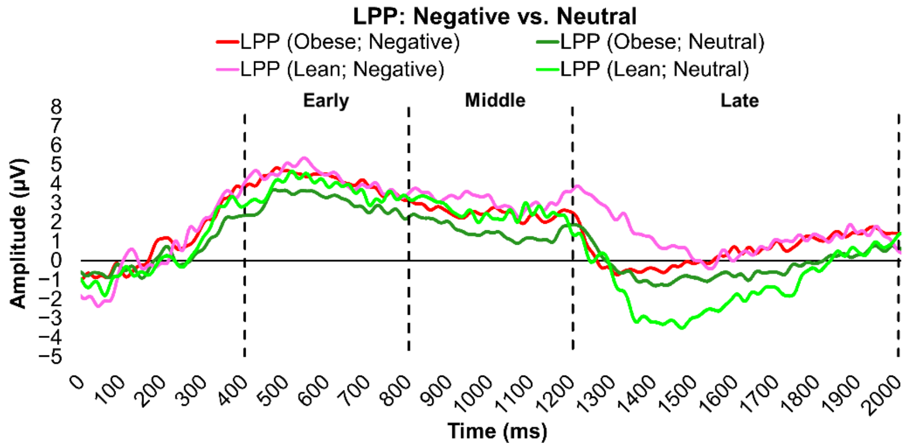
LPP: Negative vs. Neutral
LPP (Obese; Negative)
LPP (Obese; Neutral)
LPP (Lean; Negative)
LPP (Lean; Neutral)
Early
Middle
Late
Amplitude (μV)
Time (ms)

(b)

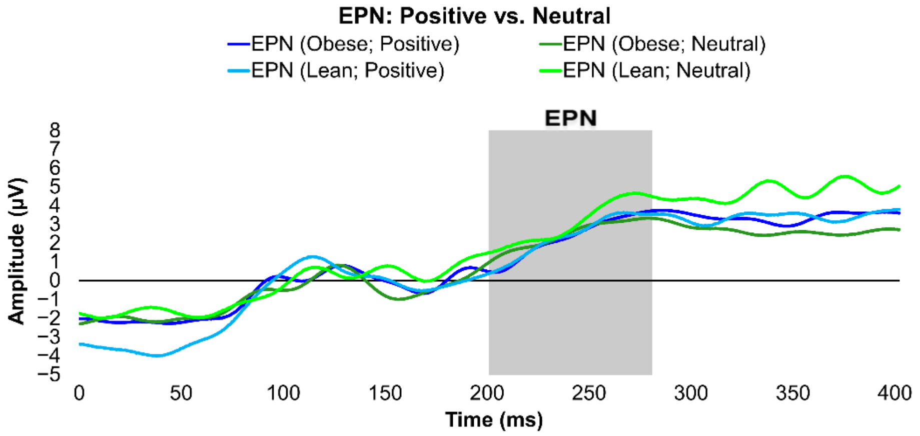
EPN: Positive vs. Neutral
EPN (Obese; Positive)
EPN (Obese; Neutral)
EPN (Lean; Positive)
EPN (Lean; Neutral)
EPN
Amplitude (μV)
Time (ms)

(c)

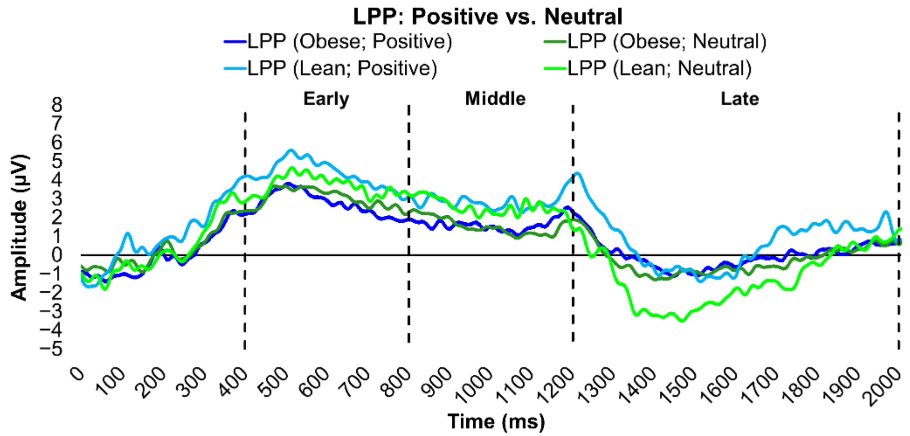
LPP: Positive vs. Neutral
LPP (Obese; Positive)
LPP (Obese; Neutral)
LPP (Lean; Positive)
LPP (Lean; Neutral)
Early
Middle
Late
Amplitude (μV)
Time (ms)

(d)

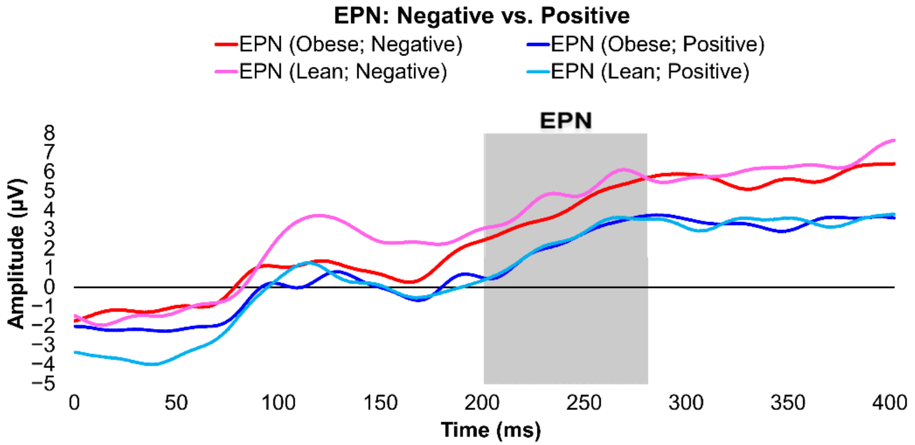

(**e**)

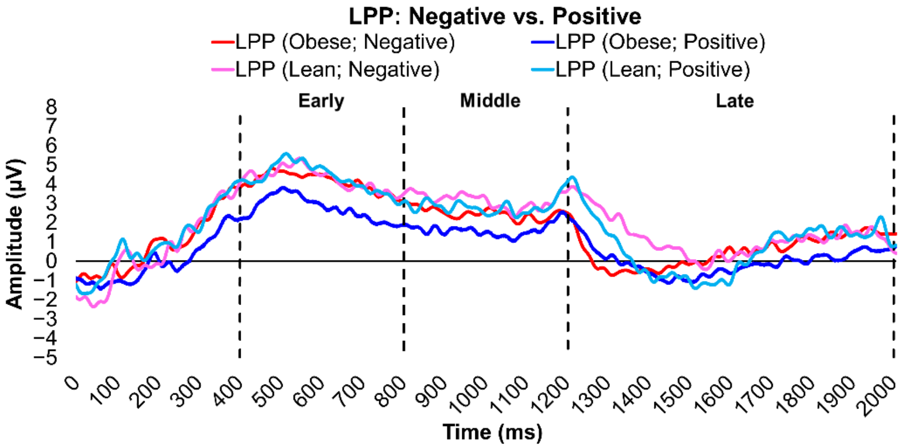

(**f**)

Supplementary Figure S2. Line graphs show the comparisons of the non-differenced grand-mean averaged EPN (left) and LPP (right) waveforms for each separate valence condition (i.e., Negative (represented by red (obese participants) or pink (lean participants) lines), Neutral (dark green (obese) or light green (lean) lines), and Positive (dark blue (obese) or light blue (lean) lines) valence conditions) that compose the contrasted picture conditions (i.e., Negative versus Neutral (**a**–**b**), Positive versus Neutral (**c**–**d**), and Negative versus Positive (**e**–**f**)) between lean (body fat percentage of <25% (males) and <35% (females)) versus obese (body fat percentage of ≥25% (males) and ≥35% (females)) subjects. In the EPN line graphs, the gray box denotes the post-stimulus period of interest for the EPN component (200–280 ms). In the LPP line graphs, the vertical, dashed lines denote the post-stimulus periods of interest for the early (400–800 ms), middle (800–1200 ms), and late (1200–2000 ms) latency windows of the LPP component. Amplitude was measured in microvolts (µV); time was measured in milliseconds. EPN = early posterior negativity; LPP = late positive potential; ms = milliseconds.

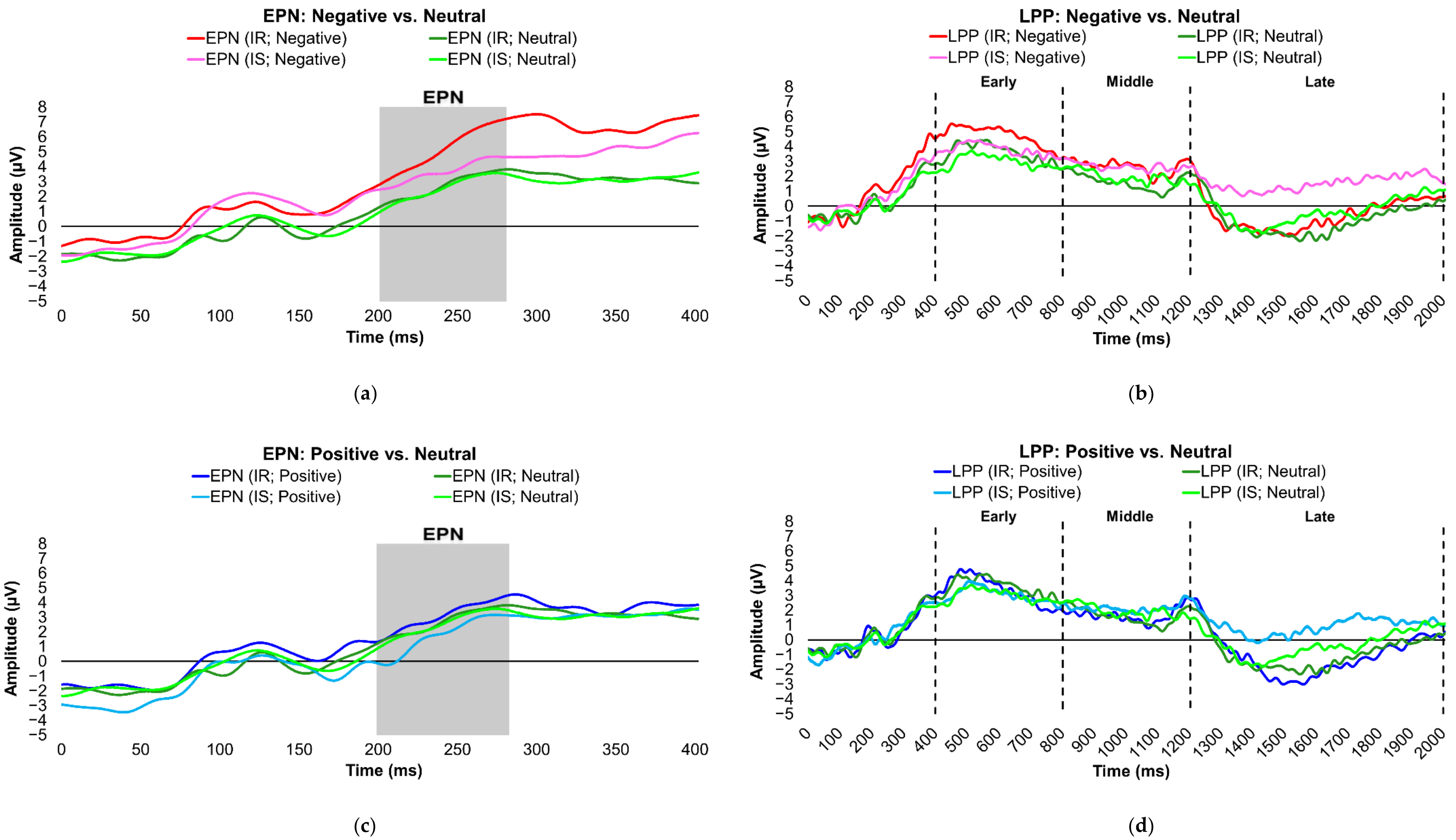
EPN: Negative vs. Neutral
EPN (IR; Negative)
EPN (IR; Neutral)
EPN (IS; Negative)
EPN (IS; Neutral)
EPN
Amplitude (μV)
Time (ms)
(a)
LPP: Negative vs. Neutral
LPP (IR; Negative)
LPP (IR; Neutral)
LPP (IS; Negative)
LPP (IS; Neutral)
Early
Middle
Late
Amplitude (μV)
Time (ms)
(b)
EPN: Positive vs. Neutral
EPN (IR; Positive)
EPN (IR; Neutral)
EPN (IS; Positive)
EPN (IS; Neutral)
EPN
Amplitude (μV)
Time (ms)
(c)
LPP: Positive vs. Neutral
LPP (IR; Positive)
LPP (IR; Neutral)
LPP (IS; Positive)
LPP (IS; Neutral)
Early
Middle
Late
Amplitude (μV)
Time (ms)
(d)

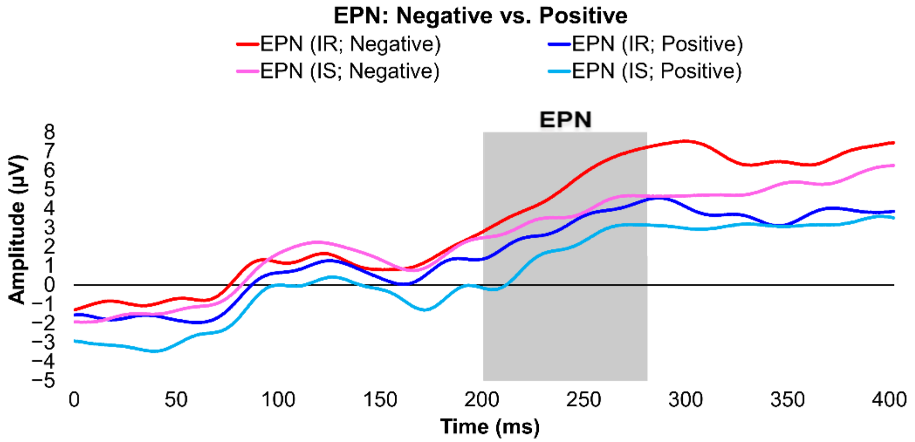

(e)

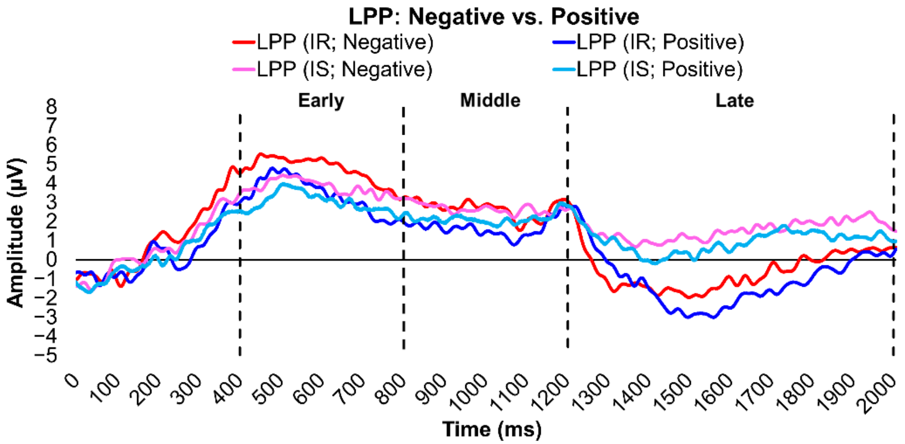

(f)

Supplementary Figure S3. Line graphs show the comparisons of the non-differenced grand-mean averaged EPN (left) and LPP (right) waveforms for each separate valence condition (i.e., Negative (represented by red (insulin-resistant participants) or pink (insulin-sensitive participants) lines), Neutral (dark green (insulin-resistant) or light green (insulin-sensitive) lines), and Positive (dark blue (insulin-resistant) or light blue (insulin-sensitive) lines) valence conditions) that compose the contrasted picture conditions (i.e., Negative versus Neutral (**a**–**b**), Positive versus Neutral (**c**–**d**), and Negative versus Positive (**e**–**f**)) between insulin-sensitive (HOMA-IR values of <2.0) versus insulin-resistant (HOMA-IR values of ≥2.0) subjects. In the EPN line graphs, the gray box denotes the post-stimulus period of interest for the EPN component (200–280 ms). In the LPP line graphs, the vertical, dashed lines denote the post-stimulus periods of interest for the early (400–800 ms), middle (800–1200 ms), and late (1200–2000 ms) latency windows of the LPP component. Amplitude was measured in microvolts (µV); time was measured in milliseconds. EPN = early posterior negativity; IR = insulin-resistant; IS = insulin-sensitive; LPP = late positive potential; ms = milliseconds.
